# Engineering an acetoacetyl-CoA reductase from *Cupriavidus necator* toward NADH preference under physiological conditions

**DOI:** 10.1038/s41598-022-07663-w

**Published:** 2022-03-08

**Authors:** Karel Olavarria, Yared O. Pijman, Ricardo Cabrera, Mark C. M. van Loosdrecht, S. Aljoscha Wahl

**Affiliations:** 1 Departement Biotechnologie, Faculteit Technische Natuurwetenschappen, Technische Universiteit Delft, Van der Maasweg 9, 2629 HZ Delft, The Netherlands; 2 Departamento de Biología, Facultad de Ciencias, Universidad de Chile, Las Palmeras 3425, Ñuñoa, Región Metropolitana Chile; 3 Present Address: Laboratory of Microbiology, Wageningen University and Research, Stippeneng 4, 6700 EH Wageningen, The Netherlands

**Keywords:** Metabolic engineering, Applied microbiology, Biocatalysis

## Abstract

The coupling of PHB generation with NADH reoxidation is required to generate PHB as a fermentation product. A fundamental trait to accomplish this feature is to express a functional NADH-preferring acetoacetyl-CoA reductase, engaged in PHB accumulation. One way to obtain such a reductase is by engineering the cofactor preference of the acetoacetyl-CoA reductase encoded by the *phaB1* gene from *Cupriavidus necator* (AAR^Cn1^). Aiming to have a deeper understanding of the structural determinants of the cofactor preference in AAR^Cn1^, and to obtain an NADH-preferring acetoacetyl-CoA reductase derived from this protein, some engineered enzymes were expressed, purified and kinetically characterized, together with the parental AAR^Cn1^. One of these engineered enzymes, Chimera 5, experimentally showed a selectivity ratio ((*k*_*cat*_/K_M_)^NADH^/(*k*_*cat*_/K_M_)^NADPH^) ≈ 18, which is 160 times higher than the selectivity ratio experimentally observed in the parental AAR^Cn1^. A thermodynamic-kinetic approach was employed to estimate the cofactor preference and flux capacity of Chimera 5 under physiological conditions. According to this approach, Chimera 5 could prefer NADH over NADPH between 25 and 150 times. Being a derivative of AAR^Cn1^, Chimera 5 should be readily functional in *Escherichia coli* and *C. necator*. Moreover, with the expected expression level, its activity should be enough to sustain PHB accumulation fluxes similar to the fluxes previously observed in these biotechnologically relevant cell factories.

## Introduction

Due to its properties, poly-3-hydroxybutyrate (PHB) could replace some fossil-fuel based plastics. A well-described PHB production pathway consists of only three reactions, catalyzed by the enzymes β-ketothiolase (E.C. 2.3.1.9), acetoacetyl-CoA reductase (E.C. 1.1.1.36) and PHB synthase (E.C. 2.3.1.304) (Supplementary Material 1). In many species, these enzymes are respectively encoded by the genes *phaA*, *phaB* and *phaC* (Fig. S1). Given its simplicity, PHB accumulation accomplished through the operation of this pathway is also a model to understand the production of other biopolymers. However, PHB production is still more expensive than fossil-fuel based plastics with similar properties. One of factors that could potentially decrease its cost is the generation of PHB as a fermentation product. However, to generate PHB as a fermentation product, PHB accumulation has to be coupled to NADH reoxidation^1,2^.

Given its high PHB accumulation titers, ability of autotrophic growth on H_2_ and CO_2_, oxygen tolerance and genetic tractability, *Cupriavidus necator* is commonly used as a platform for PHB accumulation^3^. Moreover, many of the engineered cell factories aimed at PHB production are based on the expression of the *phaCAB1* operon from *C. necator*. However, the *phaB1* gene from *C. necator* encodes for an acetoacetyl-CoA reductase, AAR^Cn1^, generally regarded as an NADPH-preferring enzyme^4,5^. Thus, to favor the NADPH-driven PHB accumulation, biomass formation is inhibited through nutrient limitation^6,7^. However, this approach could affect cellular metabolism at different levels^8^, hampering productivity^9,10^ and it requires case-specific fine-tuning of the growth conditions to properly balance biomass and PHB formations^7^.

On the other hand, if PHB production is coupled to NADH reoxidation, PHB accumulation can be generated as an anaerobic fermentation product. Although NADH reoxidation concomitant with PHB accumulation has been observed in *Azotobacter beijerinckii* and *Azotobacter vinelandii*^11,12^, kinetic characterizations of the acetoacetyl-CoA reductases from these bacteria showed that they prefer NADPH over NADH^13,14^. Other groups have claimed the observation of NADH-driven PHB accumulation using acetoacetyl-CoA reductases from *Allochromatium vinosum* or *Halomonas bluephagenesis*^1,15^. In these cases, however, the specific acetoacetyl-CoA reductase activities in cell-free extracts were measured using a single concentration of acetoacetyl-CoA and NAD(P)H, and no information about the saturation constants was provided for any of the substrates. Hence the reported results are not enough to unambiguously claim the preference for NADH of these acetoacetyl-CoA reductases, under physiological conditions.

More recently, Guedes da Silva and co-workers associated the anaerobic PHB accumulation observed in a *Candidatus* Accumulibacter phosphatis enrichment culture with the NADH-preferring acetoacetyl-CoA reductase activity observed in cell-free extracts obtained from these cells^16^. Additionally, the acetoacetyl-CoA reductase activities, at different acetoacetyl-CoA and NAD(P)H concentrations, of a protein encoded by a *phaB* gene from *Ca.* A. phosphatis, indicated that this enzyme (AAR^CAp^) has a clear preference for NADH under physiological conditions^2^. To test the in vivo functionality of AAR^CAp^ in *Escherichia coli*, a synthetic operon combining the *phaCA1* genes from *C. necator* and the cloned *phaB* gene encoding for AAR^CAp^ was assembled in a plasmid and introduced in *E. coli* cells. PHB accumulation was observed in an oxygen-limited continuous culture and, as expected, PHB titer increased with the reduction of the specific oxygen consumption rate. However, the maximum PHB accumulation titer was low^2^.

It is well-known that the reaction encoded by the β-ketothiolase (E.C. 2.3.1.9) is thermodynamically unfavorable, and evidence of physical proximity between β-ketothiolase and different enzymes catalyzing downstream reactions has been reported^17,18^. Therefore, one possible explanation for the low PHB accumulation observed in the recombinant *E. coli* cells expressing AAR^CAp^ could be a defective substrate channeling between the β-ketothiolase from *C. necator* and the acetoacetyl-CoA reductase from *Ca*. A. phosphatis due to the lack of compatible residues to establish the required protein:protein interactions. This way, if substrate channeling is indeed an important factor for the NADH-driven PHB accumulation, the ideal situation is to express the AAR^CAp^ together with the partnering β-ketothiolase from *Ca*. A. phosphatis. Nonetheless, differently than the *phaCAB1* operon from *C. necator*, AAR^CAp^ is encoded by a monocistronic *phaB* (KEGG CAP2UW1_3919; GenBank ACV37169.1), and it is not clear which are the genes from *Ca*. A. phosphatis encoding for the other enzymes partnering with AAR^CAp^ in the NADH-driven PHB accumulation observed in that organism. Additionally, in case we successfully identify those enzymes from *Ca*. A. phosphatis, we still do not know if they will be fully functional when expressed in other species.

On the other hand, the *phaCAB1* genes from *C. necator* can be functionally expressed in *E. coli* and other biotechnological relevant platforms, enabling the achievement of high PHB titers upon their expression. Hence another way to co-express a functional NADH-preferring acetoacetyl-CoA reductase with its known functional partners (the β-ketothiolase and the PHB synthase encoded by the *phaCA1* genes from *C. necator*) is turning AAR^Cn1^ into an NADH-preferring enzyme. Beyond previous kinetic studies^4,5,19^, the tridimensional structure of AAR^Cn1^ has been solved^20^ and a mutation enhancing its turnover capacity was identified in a directed evolution experiment^4^. Further, a previous segment replacement of five residues from AAR^CAp^ into the active site of AAR^Cn1^ produced the engineered enzyme Chimera 1, with an improved ability to use NADH over NADPH^2^. Considering those former results, we considered AAR^Cn1^ as a suitable starting point for protein engineering approaches envisioning the construction of an NADH-preferring acetoacetyl-CoA reductase.

## Results and discussion

### Design of the engineered acetoacetyl-CoA reductases

In a previous study, structural insights about cofactor preference in acetoacetyl-CoA reductases were obtained from comparison of the X-ray structure of AAR^Cn1^ bound to NADPH with a homology model of AAR^CAp^ in complex with NADH^2^. In the model of AAR^Cn1^, the binding pocket around the 2′-phosphate group of NADPH shows a positively charged side chain, R40, that interacts with this negatively charged phosphate group. In the case of AAR^CAp^, the positive charge of K40 could not establish this interaction because it is displaced away by the structural constraint imposed by the adjacent P41. Moreover, the bulky side chain of F38 impedes proper placement of the 2′-phosphate group in the active site. One additional feature that favors NADH binding to AAR^CAp^ is the conformation of the backbone in the stretch containing these residues. This stretch is more tightly packed in AAR^CAp^ than in AAR^Cn1^.

**Figure 1:**
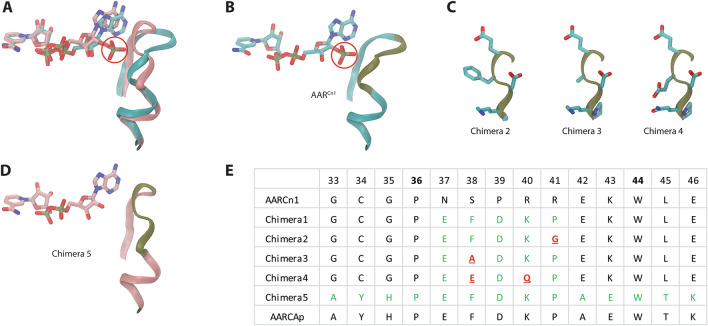
Design of the engineered versions of AAR^Cn1^. (**A**) Structural superposition of AAR^CAp^ (pink ribbon) and AAR^Cn1^ (cyan ribbon) showing the protein backbone segment surrounding the ribose moiety of NAD(P)H (residues between positions 33 and 46). The residue numeration is based on AAR^Cn1^. The characteristic 2’-phosphate of NADP(H) is highlighted with a red circle. (**B**) Individual structure of AAR^Cn1^ in association with the ribose moiety of NADPH. (**C**) Structural representation of the modifications introduced in Chimera 2, 3 and 4 with respect to Chimera 1. (**D**) In Chimera 5, the whole segment between the positions 33 and 46 in AAR^Cn1^ was replaced by the corresponding fragment from AAR^CAp^. It is possible to see that the stretch to accommodate the 2’-phosphate group is narrower in AAR^CAp^ than in AAR^Cn1^. In the backbones represented in the pictures (**B**, **C** and **D**), the segments between positions 37 and 41 were colored in dark green: this is the fragment that was modified in the Chimeras 1 to 4. (**E**) Amino acid sequences of the protein segment where modifications were introduced in the engineered enzymes. Two residues (36 and 44) are conserved in this fragment. The modified residues in the engineered enzymes are highlighted in colors, using the green color to highlight the differences with respect to the parental AAR^Cn1^, and the underlined red characters were used to highlight the changes in Chimera 2, 3 and 4 with respect to Chimera 1.

To test the predictions based on the analysis of the homology model of AAR^CAp^, an engineered enzyme, where the residues from N37 to R41 of AAR^Cn1^ were substituted by the residues E37 to P41 from AAR^CAp^ (Fig. 1), was expressed, purified and kinetically characterized^2^. This artificial enzyme, named Chimera 1, showed an increase in the selectivity ratio toward NADH ((*k*_*cat*_/K_M_)^NADH^/(*k*_*cat*_/K_M_)^NADPH^) in comparison to an NADPH-preferring acetoacetyl-CoA reductase from *C. necator* previously purified from cells of *C. necator*^19^. However, the observed selectivity ratio was 110 times lower than the selectivity constant of AAR^CAp^, and the effects of AcAcCoA concentration on the activity of Chimera 1 were not analyzed. Despite those limitations, the results obtained with Chimera 1 showed that the region of AAR^Cn1^ between N37 and R41 contains some of the structural determinants of the cofactor specificity, which is an important information toward the design of an NADH-preferring acetoacetyl-CoA reductase derived from AAR^Cn1^.

To have a deeper understanding of the role of residues F38, K40 and P41 in the kinetic properties of Chimera 1, we designed some engineered enzymes. (i) The mutation P41G (Chimera 2) was intended to test the importance of the conformational restraints imposed by proline. Glycine (G) has the smallest side chain among the proteinogenic amino acids; therefore, it should be possible to see the effects of removing the side chain of the residue P41 without introducing a large side chain. (ii) The mutation F38A (Chimera 3) was generated with the purpose of evaluating the absence of a bulky side chain while maintaining a nonpolar lateral chain (Alanine). (iii) The double mutant F38E K40Q (Chimera 4) was designed to replace the bulky nonpolar side chain of F38 by a side change of a similar size but carrying a negative charge (Glutamic acid). This way, the side chain of E38 could establish a hydrogen bond with the ribose of the adenosine moiety of NADH. Simultaneously, the positive charge of K40 was replaced by a residue with a side chain of similar size (Glutamine) but without charge to avoid the formation of a saline bridge between E38 and K40 that could hinder the expected interaction between E38 and NADH (Fig. 1).

Aiming for a backbone conformation of the β2α2 loop more akin to that observed in the model of AAR^CAp^, we decided to replace in AAR^Cn1^ the whole segment between G33 and E46 by the corresponding residues from AAR^CAp^ (Fig. 1). This fragment replacement approach was already employed in the construction of Chimera 1, and it is similar to the approach employed by Yaoi and co-workers to modify the cofactor preference of the isocitrate dehydrogenase (E.C. 1.1.1.42) from *Thermus thermophiles*^21^. In this latter case, the cofactor preference of this enzyme was shifted from NADP^+^ to NAD^+^ by replacing an 8-residue segment by the corresponding sequence from an isopropyl-malate dehydrogenase (E.C. 1.1.1.85). Therefore, we think that replacing a bigger segment could represent an advantage over the site-directed approach, because it can better recreate the interactions occurring in the NADH-specific site.

Overall, we designed four engineered enzymes: three carrying single or double residue substitutions with respect to Chimera 1 with the aim of better understanding the roles of some residues in this engineered enzyme, and a fourth engineered protein where a fragment of 13 residues from AAR^Cn1^ were substituted by the corresponding residues from AAR^CAp^.

### Experimental characterization of AAR^Cn1^ and the engineered acetoacetyl-CoA reductases

Both the parental AAR^Cn1^ and the engineered enzymes were purified from recombinant *E. coli* BL21DE3 strains expressing these proteins. The kinetic characterization of these enzymes was performed by reaction progress curve analysis (see Supplementary Material 5). Aiming to have a more accurate comparison of the engineered enzymes with the parental protein, we estimated K_M_^NADH^, K_M_^NADPH^ and the apparent turnover constants for all these enzymes under similar experimental conditions: AcAcCoA 2 mM and NAD(P)H varying between 10 and 600 µM. Under these experimental conditions, the model that best explained the observations was the simple Michaelis–Menten.

We found that AAR^Cn1^ has a higher catalytic efficiency with NADPH (0.118 µM/s) than with NADH (0.013 µM/s) (Table 1), which is consistent with previous observations where acetoacetyl-CoA reductase activities in *C. necator* were measured with both NADH and NADPH^19,22^. If the velocities of the reactions using NADH or NADPH are calculated with the simple Michaelis–Menten equation (Supplementary Material 7), these velocities are very similar when NAD(P)H concentration is 650 µM (Fig. S8). However, substrate inhibition caused by AcAcCoA have been previously observed^5,19^. We also detected evidence of substrate inhibition: using AcAcCoA 2 mM and NADPH varying between 10 and 200 µM, we observed a *k*_*cat*_^NADPH^ = 5.2 s^−1^ while Zhang and co-workers^5^ observed a *k*_*cat*_^NADPH^ = 71 s^−1^ using AcAcCoA concentration = 20 µM.

**Table 1:** Experimentally observed kinetic parameters obtained varying the concentration of NADH or NADPH, at a fixed initial concentration of AcAcCoA.

	AAR^Cn1^	Chimera1^a^	Chimera2	Chimera3	Chimera4	Chimera5
*k*_*cat*_ (NADH) (s^−1^)	10.3 [9.1–11.6]	5.1	4.1 [3.8–4.4]	19.5 [17.8–21.9]	12.2 [10.5–14.8]	10.5 [10.3–10.7]
K_M_ (NADH) (µM)	819 [688–975]	77	140 [126–158]	886 [770–1037]	618 [488–814]	43 [38–50]
*k*_*cat*_ (NADPH) (s^−1^)	5.2 [5.1–5.4]	9	13.1 [9.6–21.3]	29 [23–36]	3.1 [2.8–3.6]	38.4 [28.2–57.9]
K_M_ (NADPH) (µM)	44 [40–49]	665	1117 [789–1864]	1752 [1329–2275]	120 [84–168]	2787 [1931–4382]
(*k*_*cat*_/K_M_)^NADH^ (µM/s)	0.013	0.066	0.029	0.022	0.020	0.244
(*k*_*cat*_/K_M_)^NADPH^ (µM/s)	0.118	0.014	0.012	0.017	0.026	0.014
selectivity ratio^b^	0.11	4.89	2.50	1.33	0.76	17.72

For comparison, some reference data from literature are included. Parameter estimates are represented as best-fitted values and their 95% confidence intervals between squared brackets.

^a^Data obtained from Olavarria and co-workers^2^. ^b^Selectivity ratio defined as (*k*_*cat*_/K_M_)^NADH^/(*k*_*cat*_/K_M_)^NADPH^.

If we assume that (i) substrate inhibition is caused by the binding of a second molecule of AcAcCoA to the enzyme-AcAcCoA complex and (ii) K_i_^AcAcCoA^ ≈ K_M_^AcAcCoA^ = 2 µM^5^, it is possible to calculate the inhibition constant (K_iS_^AcAcCoA^), solving the equation IX-388 from Segel^23^:

1 $${k}_{cat}^{app (2mM)}=\frac{{k}_{cat}^{app \left(20 \, \mathrm{\mu M}\right)}*AcAcCoA *NADPH}{{K}_{i}^{AcAcCoA}*{K}_{M}^{NADPH}+{K}_{M}^{AcAcCoA}*NADPH +{K}_{M}^{NADPH}*AcAcCoA*\left(1+\frac{AcAcCoA}{{{\varvec{K}}}_{{\varvec{i}}{\varvec{S}}}^{{\varvec{A}}{\varvec{c}}{\varvec{A}}{\varvec{c}}{\varvec{C}}{\varvec{o}}{\varvec{A}}}}\right)+AcAcCoA*NADPH}$$

$$5.2 {s}^{-1}=\frac{71 {s}^{-1}*2000 \,  \upmu {\text{M}}*200 \,  \upmu {\text{M}}}{2  \, \upmu {\text{M}}*44  \, \upmu {\text{M}}+2  \, \upmu {\text{M}}*200  \, \upmu {\text{M}}+44  \, \upmu {\text{M}}*2000  \, \upmu {\text{M}}*\left(1+\frac{2000 \,  \upmu {\text{M}}}{{{\varvec{K}}}_{{\varvec{i}}{\varvec{S}}}^{{\varvec{A}}{\varvec{c}}{\varvec{A}}{\varvec{c}}{\varvec{C}}{\varvec{o}}{\varvec{A}}}}\right)+2000 \,  \upmu {\text{M}}*200  \, \upmu {\text{M}}}$$

Solving, the value K_iS_^AcAcCoA^ = 35 µM is obtained. If our assumptions are correct, it should be possible to calculate the critical AcAcCoA concentration allowing the maximum velocity (AcAcCoA^Vmax^), using the equation:

2 $${AcAcCoA}^{Vmax}=\sqrt[2]{{K}_{i}^{AcAcCoA}*{K}_{iS}^{AcAcCoA}*(1+\frac{{K}_{M}^{AcAcCoA}*NADPH}{{K}_{i}^{AcAcCoA}*{K}_{M}^{NADPH}})}\approx 9  \, \upmu {\text{M}}$$

Indeed, this AcAcCoA^Vmax^ is very similar to the AcAcCoA concentration = 12 µM at which Zhang and co-workers registered the maximum velocity^5^. Doing a similar analysis but using the estimates K_M_^AcAcCoA^ = 5.7 µM and *k*_*cat*_^NADPH^ = 102 s^−1^ observed by Matsumoto and co-workers^4^, the values K_iS_^AcAcCoA^ = 24 µM and AcAcCoA^Vmax^ = 17 µM can be obtained, which are also very similar to the results obtained by Zhang and co-workers.

On the other hand, Haywood and co-workers characterized an NADPH-preferring acetoacetyl-CoA reductase directly purified from cells of *C. necator* (known as *Alcaligenes eutrophus* at that time). This enzyme had a molecular weight of 23 kDa, it “showed significant (20%) activity with NADH”, and participated in PHB synthesis^19^. For the NADPH-driven reaction, Haywood and co-workers observed a K_M_^AcAcCoA^ = 5 µM and an AcAcCoA^Vmax^ = 32 µM. With these data, it is possible to write

$${AcAcCoA}^{Vmax}=32 \,  \upmu {\text{M}}=\sqrt[2]{5  \, \upmu {\text{M}}*{{\varvec{K}}}_{{\varvec{i}}{\varvec{S}}}^{{\varvec{A}}{\varvec{c}}{\varvec{A}}{\varvec{c}}{\varvec{C}}{\varvec{o}}{\varvec{A}}}* \left(1+\frac{5  \, \upmu {\text{M}}*200  \, \upmu {\text{M}}}{5  \, \upmu {\text{M}}*44 \,  \upmu {\text{M}}}\right)}$$

and to find the solution K_iS_^AcAcCoA^ ≈ 37 µM, which is also similar to the K_iS_^AcAcCoA^ estimated with the kinetic parameters obtained by other research groups. Therefore, it seems that the NADPH-preferring acetoacetyl-CoA reductase studied by Haywood and co-workers was also the protein encoded by *phaB1*.

Overall, the substrate inhibition pattern observed in AAR^Cn1^ by several groups is consistent with an ordered mechanism where AcAcCoA binds first to the enzyme, and a second molecule of AcAcCoA can also bind to the enzyme-AcAcCoA complex, causing the observed substrate inhibition.

Regarding the engineered enzymes Chimera 2 and Chimera 3, their catalytic efficiencies using NADPH and NADH were similar, indicating a loss in the discrimination capacity at low cofactor concentrations (Table 1). The catalytic efficiencies using NADPH of these enzymes were very similar to Chimera 1, indicating that, contrary to our original hypothesis, P41 and F38 are not the key structural elements to discriminate against NADPH. On the other hand, the changes F38E and K40Q introduced in Chimera 4 also meant a loss in the cofactor discrimination ability of Chimera 4 at low NAD(P)H concentrations (Table 1). Nevertheless, its selectivity for NADH increases at higher cofactor concentrations (Fig. S8). This property could be biotechnologically relevant for the NADH-driven PHB production because it is known that the NADH/NAD^+^ ratio increases in oxygen-limiting conditions, both in *E. coli* and *C. necator*^24,25^. In the case of Chimera 5, we observed an increase in the catalytic efficiency using NADH, driven by an increase in *k*_*cat*_^NADH^ and a decrease in K_M_^NADH^ with respect to Chimera 1 (Table 1). At the same time, the catalytic efficiency using NADPH remained similar. These results suggest that (i) the fragment of AAR^CAp^ between the residues A33 and K46 contains important structural determinants for its preferential use of NADH, and (ii) that some structural elements hindering the reactions with NADPH in AAR^CAp^ are outside this fragment.

Overall, Chimera 5 showed the highest selectivity ratio among the engineered enzymes (3.6 times larger than the selectivity ratio observed in Chimera 1, Table 1). However, the estimates of *k*_*cat*_^NAD(P)H^ and K_M_^NAD(P)H^ reported in Table 1 were obtained with AcAcCoA 2 mM. Considering that inhibition by AcAcCoA has been previously reported, we decided to evaluate the effects of AcAcCoA concentration on the rates of the reactions catalyzed by Chimera 5.

**Figure 2:**
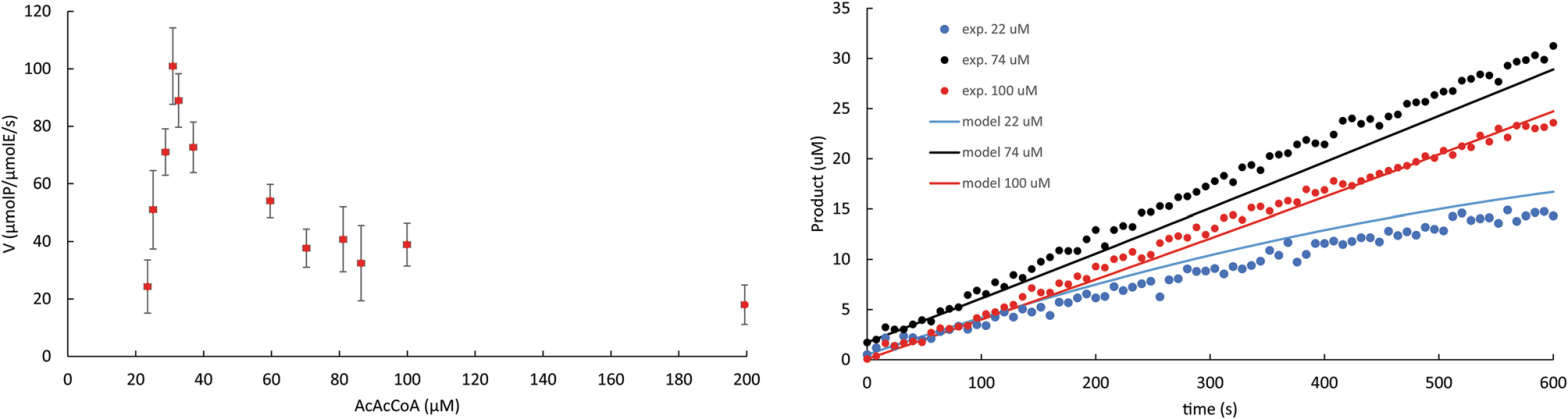
Experimental observation of the AcAcCoA inhibition of the reactions catalyzed by Chimera 5, using NADH as cofactor. Left side: experimentally determined initial rates, at different initial concentrations of AcAcCoA. The error bars represent the standard deviations obtained during the calculation of the slopes Δ[Product]/Δtime. Right side: selection of three experimental progress curves (exp.), at three different initial concentrations of AcAcCoA (22, 74 and 100 µM). The continuous curves represent the global fitting using a substrate inhibition model where K_iS_ = K_M_.

Initial AcAcCoA concentration was varied between 5 and 200 µM while keeping the initial NAD(P)H concentration at 1 mM. Substrate inhibition was observed in the reactions using NADH and NADPH. In the reactions using NADH, the inhibitory effect of AcAcCoA was best explained by a model where the substrate inhibition constant (K_iS_) is equal to K_M_ (Fig. 2; Table 2). It is worth noting that, because of substrate inhibition, the turnover constants obtained varying AcAcCoA concentrations (Table 2) were higher than the turnover constants obtained varying NAD(P)H concentrations (high AcAcCoA concentration) (Table 1).

**Table 2:** Experimentally observed kinetic parameters of the reactions catalyzed by Chimera 5, obtained varying the concentration of AcAcCoA, at a fixed initial concentration of NADH or NADPH.

	NADH	NADPH
*k*_*cat*_ (s^−1^)	147 [144–151]	76 [66–95]
K_M_^AcAcCoA^ (µM)	50 [46–53]	48 [36–71]
K_iS_^AcAcCoA^ (µM)	K_iS_ = K_M_^a^	324 [195–465]

Parameter estimates are represented as best-fitted value and their 95% confidence intervals between squared brackets.

^a^In the model that best explained the experimental results, the substrate inhibition constant (K_iS_) was equal to the K_M_.

### Modeling the flux capacity and cofactor preference under physiological conditions

Given the standard Gibbs free energy of the reaction catalyzed by β-ketothiolase [Δ_r_G° = 25.0 ± 1.7 kJ/mol (eQuilibrator^26^)] and the observed physiological concentrations of acetyl-CoA (0.2 mM–2 mM^27^) and coenzyme A (88 µM–63 mM^27^), the reaction catalyzed by β-ketothiolase will be thermodynamically feasible only at very low AcAcCoA concentrations. At such low AcAcCoA concentrations, it is not clear what could be the physiological role of the inhibition of AAR^Cn1^ by AcAcCoA. However, these low AcAcCoA concentrations should have a high impact on the rate of the reactions catalyzed by AAR^Cn1^ or Chimera 5. On the other hand, NADH/NAD^+^ ratios can change, depending on the external redox state^24^; and NADPH/NADP^+^ ratios can also change depending on oxidative stress or nutrient limitation^28^. This way, the in vivo cofactor preference cannot be characterized by a single number. Instead, a more realistic estimation of the relative use of NADH over NADPH requires considering the dynamics of the in vivo concentrations. Therefore, we combined thermodynamic and kinetic analyses (thermodynamic-kinetic approach) to model the flux capacity and cofactor preference of Chimera 5 under physiological conditions. Aiming to have reference values for these quantifications, the same approach was applied to the parental enzyme AAR^Cn1^. The MATLAB code for these calculations is provided as an Appendix in the Supplementary Materials.

The maximum metabolic flux that Chimera 5 can sustain (flux capacity) and its relative use of NADH over NADPH will depend on the values of some (kinetic and thermodynamic) constants and some variable (enzymes, substrates and products) concentrations. The cytoplasmic concentration of Chimera 5 will depend on the gene expression system and the cellular conditions: from a chromosomal or an episomal locus, gene copy number, promoters, codon usage, ammonium availability, etcetera. As a first approximation, it is possible to analyze a scenario where the engineered *phaB*^*Chimera5*^ gene replaces the parental *phaB1* gene. In that case, using the specific acetoacetyl-CoA reductase activity measured in a cell-free extract obtained from *C. necator* cells expressing only the paralog *phaB1*^22^, we can estimate a cytoplasmic enzyme concentration of 428 µmol/L_cytoplasm_ (see detailed calculation in Supplementary Material 8). Having the cytoplasmic enzyme concentration, it is possible to calculate the expected NADH- and NADPH-driven forward flux capacities (*J*^*forward*^), evaluating the following equation with the kinetic parameters of Chimera 5, and the cytoplasmic concentrations of NAD(P)H and AcAcCoA:

3 $${J}^{forward}=\frac{E*{k}_{cat}*AcAcCoA *NAD(P)H}{{K}_{i}^{AcAcCoA}*{K}_{M}^{NAD(P)H}+{K}_{M}^{AcAcCoA}*NAD(P)H +{K}_{M}^{NAD(P)H}*AcAcCoA*\left(1+\frac{AcAcCoA}{{K}_{iS}}\right)+AcAcCoA*NAD(P)H}$$

However, under physiological conditions, the net metabolic capacity is also affected by the presence of other metabolites interacting with the enzyme catalyzing the reaction. For the sake of simplicity, we just focused in the quantification of the reverse reaction. Although in this research we did not investigate the kinetic parameters for the backward reactions, these parameters are not independent of the kinetic parameters of the forward reactions: they are linked through the thermodynamic equilibrium constant (Haldane relationships)^23,29^. This way, a generalized equation to calculate the expected backward flux (*J*^*backward*^) is the flux force relationship^30^:

4 $${J}^{backward}= {J}^{forward}*{e}^{\frac{{\Delta }_{r}G}{RT}}$$

Both for the calculation of the forward flux capacities and the Gibbs free energies (required to calculate the backward fluxes), it is necessary to have a reliable estimation of the cytoplasmic concentrations of the involved metabolites. In the Supplementary Material 8 it is possible to find an explanation of the method here employed to estimate the cytoplasmic concentrations of NAD(P)H, AcAcCoA, coenzyme A and 3-hydroxybutyryl-CoA.

**Figure 3:**
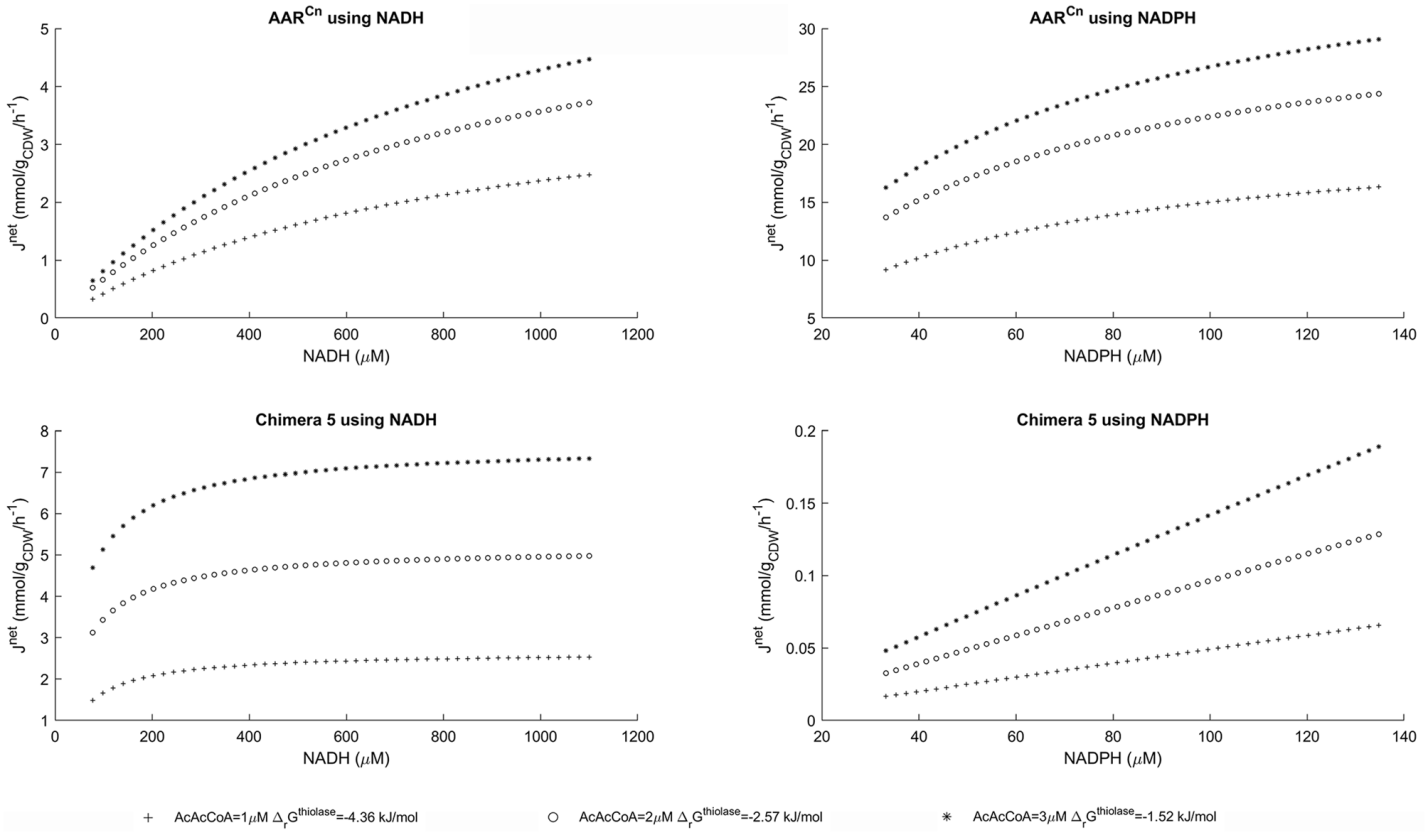
Flux capacities (J^net^) of AAR^Cn1^ and Chimera 5 at different AcAcCoA, NADH and NADPH concentrations, calculated using a thermodynamic-kinetic approach. Flux capacities were calculated as the differences between forward and backward fluxes. Forward fluxes were calculated evaluating a rate equation [(Eq. (3)] with suitable enzyme and substrate concentrations; and backward fluxes were estimated using the Flux-Force relationship [(Eq. (4)] (see the text and Supplementary Material 8 for details). The tested metabolites concentrations enable that the condensation of two molecules of acetyl-CoA (catalyzed by the β-kethothiolase) and the reduction of AcAcCoA with NAD(P)H (catalyzed by the acetoacetyl-CoA reductases) are thermodynamically feasible. It is possible to notice that the higher the AcAcCoA concentrations, the higher are the flux capacities of the acetoacetyl-CoA reductases, but the Gibbs free energies of the reaction catalyzed by the β-kethothiolase are lower.

**Figure 4:**
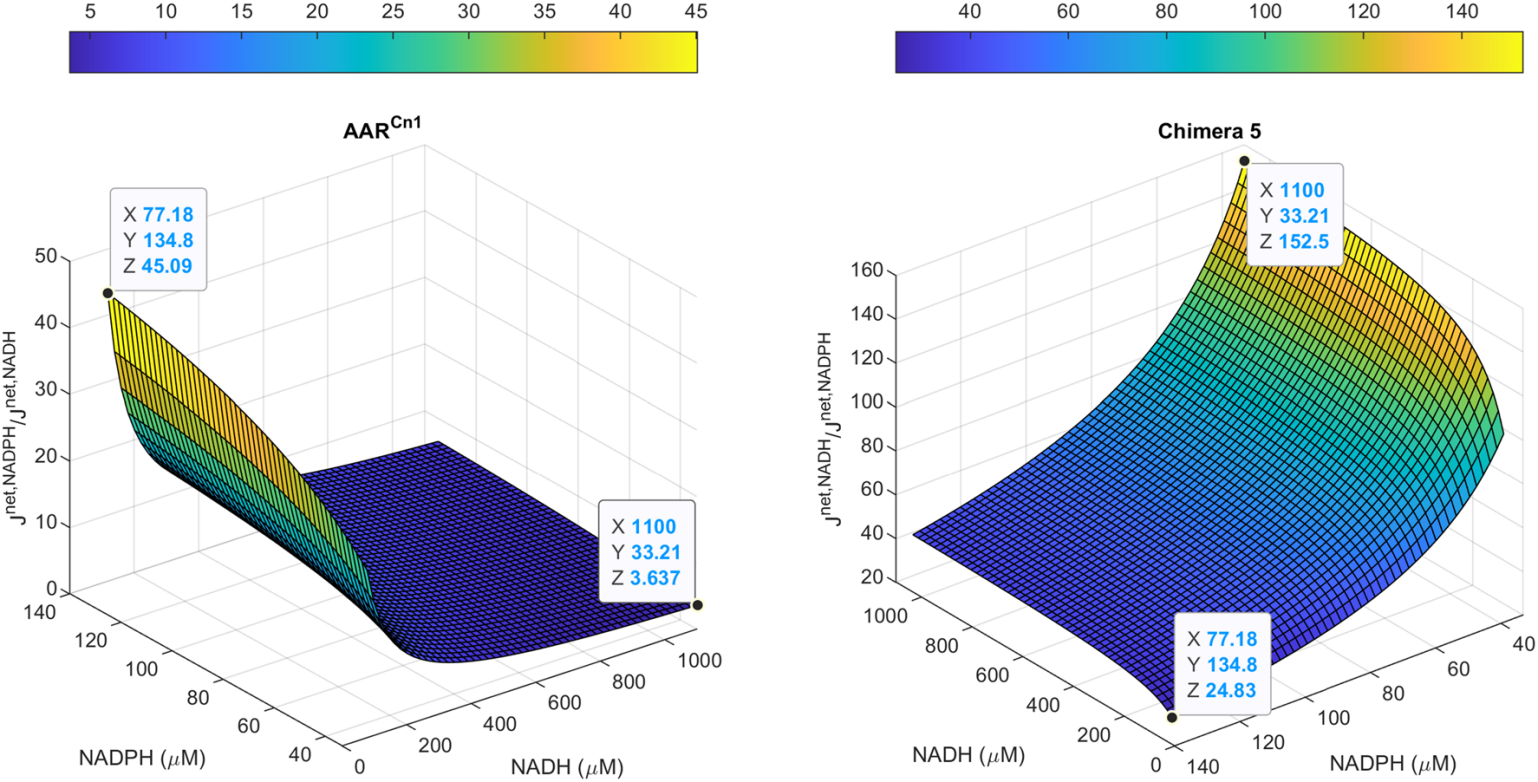
Cofactor preferences of AAR^Cn1^ and Chimera 5 at different combinations of NADH and NADPH concentrations. Cofactor preferences were calculated as the ratios between the flux capacities obtained for each cofactor. In the case of AAR^Cn1^, the preferences were calculated as the ratios of the flux capacities obtained for NADPH over the flux capacities obtained for NADH. In the case of Chimera 5, the preferences were calculated as the ratios of the flux capacities obtained for NADH over the flux capacities obtained for NADPH. The values of the minimum and maximum cofactor preferences (Z), and the values of NADH (X) and NADPH (Y) at which these extremes cofactor preferences values were obtained are highlighted in the graph.

This way, the metabolic flux capacities (*J*^*net*^) of AAR^Cn^ and Chimera 5 were calculated as *J*^*net*^ = *J*^*forward*^ – *J*^*backward*^ (Fig. 3), and the relative uses of NADH over NADPH by these enzymes were calculated as the ratios *J*^*net(NADH)*^/*J*^*net(NADPH)*^ (Fig. 4). The flux capacities and the relative use of NADH over NADPH were obtained for the different combinations of NAD(P)(H) concentrations. Different observations can be discussed from these results.

Du and co-workers^31^ observed a specific PHB production rates (q_PHB_) of 0.14 mmol^PHB^/mmolX/h = 5.8 mmol^PHB^/g_CDW_/h in an ammonium-limited continuous culture of this bacterium. Therefore, the metabolic flux capacity calculated for AAR^Cn1^ using NADPH should be enough to sustain the experimentally observed q_PHB_ (Fig. 3). Moreover, our calculations of the flux capacity of AAR^Cn1^ using NADPH are consistent with a previous observations pointing to *phaB1* as the paralog with the main role in PHB accumulation in *C. necator*^22^. Regarding Chimera 5, our calculations show that although its flux capacity using NADH is lower than the flux capacity of AAR^Cn1^ using NADPH, it should be enough to sustain NADH-driven q_PHB_ of up to 7 mmol/gCDW/h (Fig. 3). This analysis also shows that the flux capacities of Chimera 5 are more influenced by AcAcCoA concentrations than the respective flux capacities of AAR^Cn1^. This can be explained by the relatively high K_M_^AcAcCoA^ values of Chimera 5 ([AcAcCoA]^physiological^ << K_M_^AcAcCoA^). Further protein engineering efforts could help to decrease the value of K_M_^AcAcCoA^(NADH). In addition, more studies are required to determine whether the AcAcCoA inhibition has or has not a physiological role.

Regarding the relative use of NADH over NADPH, our calculations indicate that Chimera 5 will prefer NADH over NADPH (Fig. 4); and this preference can be up to 150 times at larger NADH/NAD^+^ ratios (for example, under anaerobic conditions^24^). It should be noticed that the estimated preferences for NADH calculated using this thermodynamic-kinetic approach are different (higher) than the selectivity ratio (*k*_*cat*_/K_M_)^NADH^/(*k*_*cat*_/K_M_)^NADPH^ ≈ 18. We think that the cofactor preferences obtained with the thermodynamic-kinetic approach are physiologically more relevant because these calculations include the effects of AcAcCoA and product concentrations.

Using the same approach, it is possible to see that although AAR^Cn1^ prefers NADPH over NADH, this preference could decrease drastically when NADPH concentration is low and NADH concentration is high (Fig. 4). This dual cofactor preference of AAR^Cn1^ has been previously shown^19,22,32^; and Haywood and co-workers related this trait to the generation of both NADH and NADPH in the Entner–Doudoroff pathway^19^, which is the main source of reducing power when *C. necator* is growing on sugars.

Finally, one note of caution is required: the results obtained with this thermodynamic-kinetic approach can be affected by different factors, mainly AcAcCoA concentrations. The higher the AcAcCoA concentration, the higher is the capacity of the acetoacetyl-CoA reductase but the lower is the thermodynamic driving force for the reaction catalyzed by β-ketothiolase (Fig. 3). According to our calculations, there is a narrow concentration window (between 1 and 3 µM) where both acetoacetyl-CoA reductase and β-ketothiolase are thermodynamically feasible. This thermodynamic bottleneck could be released if substrate channeling is established these enzymes. Further studies are required to see if substrate channeling, as described in similar systems^18^, is present or not in this case.

### Biotechnological relevance of an NADH-preferring acetoacetyl-CoA reductase derived from AAR^Cn1^

There are many examples of successful expression of the *phaCAB1* genes from *C. necator* in *E. coli*, resulting in PHB accumulations of more than 80% of the cell dry weight^33^. The possibility of rewiring the glycolytic pathways of *E. coli* to enhance the supply of acetyl-CoA^34^, combined with the suppression of competing by-products, and the expression of the *phaCAB*^*Chimera5*^ operon should enable a more efficient generation of PHB as a fermentation product. Considering that the genetic tools to modify *C. necator* are available, it should be readily possible to replace the chromosomal copy of *phaB1* by *phaB*^*Chimera5*^. In *C. necator*, the reaction catalyzed by glucose-6-phosphate dehydrogenase has been identified as the main source of NAD(P)H during the sugar-driven PHB accumulation. Replacement of the native glucose-6-phosphate dehydrogenase by an NAD^+^-preferring glucose-6-phosphate dehydrogenase^35^ should thus improve the match between the catabolic supply and the PHB production demand of NADH, without enforcing the inhibition of biomass formation.

Overall, given its preference for NADH, the expression of Chimera 5 should result in engineered *E. coli* or *C. necator* strains with enhanced capacities to accumulate PHB under oxygen-limiting conditions. These oxygen-limiting conditions have clear process and economic advantages over the traditional fully aerobic PHB production process^36^. Moreover, given the previously observed functionality of AAR^Cn1^ in different species, construction of an NADH-preferring acetoacetyl-CoA reductase derived from AAR^Cn1^ should enable the generation of PHB as a fermentation product in platforms such as *C. necator*, *E. coli*, *Corynebacterium glutamicum* or *Saccharomyces cerevisiae*, using carbon-plus-electron sources as diverse as hexoses, pentoses, alcohols, or syngas.

## Methods

### DNA manipulations and protein purification

Artificial DNA sequences encoding for the amino acid sequences of the engineered enzymes were ordered at Integrated DNA Technologies (IDT, Belgium). The corresponding DNA sequences are reported in Supplementary Material 2. Routine DNA manipulations were performed according to standard procedures described elsewhere^37^. Protein purification was performed by immobilized metal affinity chromatography (IMAC) (Fig. S2). More details about DNA manipulations and protein purification are provided in Supplementary Material 2.

### Experimental design for the enzymatic assays

Preliminary estimations of the kinetic parameters using the simple Michaelis–Menten model showed poor fitting (Figs. S5 and S6). To overcome this problem, we assessed the kinetic parameters through the reaction progress curve analysis. A simulation tool to support the design of the kinetic assays using reaction progress curves was developed (Fig. S4). Briefly, the developed simulation tool enables to study the impact of enzyme saturation, thermodynamic driving force, enzyme catalytic power, time length of the data recording window, data acquisition rate, and experimental error on the accuracy of the kinetic parameter estimations. It was specifically designed to explore, in silico, the differences between the *true* kinetic parameters and the *best-fitted* kinetic parameters obtained assuming the simple Michaelis–Menten model. Guided by this simulation tool, we chose the experimentally suitable conditions to obtain *k*_*cat*_ and K_M_ from the analysis of reaction progress curves catalyzed by acetoacetyl-CoA reductases (Fig. S7). A detailed explanation about the fundamentals of this simulation tool can be found in the Supplementary Material 5. The MATLAB code for these calculations is provided as an Appendix in the Supplementary Materials.

### Enzyme kinetics assays

Kinetic assays were performed in MOPS buffer (((3-(N-morpholino)-propanesulfonic acid) 50 mM, NaCl 5 mM, MgCl_2_ 5 mM, pH 7.0)) at 30 °C in a Synergy HTX plate reader (Biotek) using 96 wells half-area microplates (Greiner, code 675,101). The enzyme concentration during the enzymatic assays was 2 nM (Fig. S3). Substrate stocks were always freshly prepared from reagents with analytical grade (purchased from Santa Cruz Biotechnology or Sigma-Aldrich). The substrate concentrations in such stocks were estimated by spectrophotometry, applying the Lambert–Beer law (Supplementary Material 3). Changes in substrate concentrations were followed by spectrophotometry, observing the changes in absorbance at 340 nm or 360 nm. Because both AcAcCoA and NAD(P)H have a sizable absorbance at 340 nm and 360 nm, calculation of the variation of product concentration in time required special attention (Supplementary Material 4).

### Statistical analysis

To evaluate the suitability of different kinetic models to describe the experimental observations, we performed model discrimination analyses with the software DYNAFIT (Biokin)^38^. The following models were considered for the experiments where the NAD(P)H concentrations were varied while keeping constant the initial AcAcCoA concentration: (i) simple Michaelis–Menten, (ii) competitive product inhibition, (iii) non-competitive product inhibition, and (iv) mixed product inhibition. In the reactions where the initial AcAcCoA concentration was varied at a fixed NAD(P)H concentration, the following models were considered: (i) simple Michaelis–Menten, (ii) typical substrate inhibition, (iii) substrate inhibition with K_iS_ = K_M_, (iv) substrate plus product inhibition, and (v) mixed inhibition with an inactive ESS complex. The molecular interactions considered for each one of these models are shown in Supplementary Material 6.

During the model discrimination analyses, the adjustment of the different kinetic models under comparison to the experimental data was evaluated in the following time windows along the reaction progress curves: 5, 10, 15, 20 and 25 min. Next, for the assessment of the kinetic parameters we chose the kinetic model and the time window with the narrowest empirical coefficient of variation (CV_e_). For the chosen model and time window, the best fitted estimates with their associated 95% confidence intervals were determined using a Monte Carlo algorithm (1000 runs), built into the DYNAFIT software. CV_e_ are calculated by DYNAFIT as an indicator of dispersion for the parameters obtained in continuous assays. The different experimental points in continuous assays are not statistically independent from each other, therefore the typical standard errors are not a correct indicator of dispersion. The CV_e_ were calculated as follows:

5 $${CV}_{e}=100*\frac{SE}{\widehat{p}}*\sqrt{\frac{{n}_{D}/({n}_{P}-1)}{R-1}}$$

where *CV*_*e*_ is the empirical coefficient of variation, *SE* is the standard error, $\widehat{p}$ is the parameter best-fitted value, *n*_*P*_ is the number of adjustable parameters in the model, *n*_*D*_ is the number of experimental data points, and *R* is a control parameter expressing how much the typical coefficient of variation is “inflated” with the introduced correction. *R* was set to a value of 10.

## Supplementary Information

Supplementary Information 1.

Supplementary Information 2.

## Data Availability

All the raw experimental data, Microsoft Excel calculation datasheets, MATLAB scripts and DYNAFIT scripts employed in this research project are openly available at the Figshare database (figshare.com), under 10.6084/m9.figshare.16613794 and 10.6084/m9.figshare.19169321.v1.
