# Supplementary material for: Engineering an acetoacetyl-CoA reductase from Cupriavidus necator toward NADH preference under physiological conditions
Source: Sci Rep. 2022 Mar 8;12:3757. doi: 10.1038/s41598-022-07663-w (PMC8904767; doi:10.1038/s41598-022-07663-w)
Supplement: Supplementary file 1 — Supplementary Information 1. [file 41598_2022_7663_MOESM1_ESM.docx]

Supplementary materials

[Supplementary Material 1: PHB generation pathway. 2](#_Toc95732077)

[Supplementary Material 2: DNA manipulations and protein purification 2](#_Toc95732078)

[Supplementary Material 3: Quantification of the reagents 6](#_Toc95732079)

[Supplementary Material 4: Quantification of product concentration during the enzymatic assays 9](#_Toc95732080)

[Supplementary Material 5: A simulation tool to evaluate the accuracy of the kinetic parameters assessed by reaction progress curves analysis 11](#_Toc95732081)

[Supplementary Material 6: Biochemical mechanisms under consideration 18](#_Toc95732082)

[Supplementary Material 7: Relative use of NADH over NADPH at high AcAcCoA concentration 21](#_Toc95732083)

[Supplementary Material 8: Calculating the cytoplasmic acetoacetyl-CoA reductase and metabolites concentrations 22](#_Toc95732084)

[Supplementary References 24](#_Toc95732085)

[Appendix 1: MATLAB code of the Simulation tool to evaluate accuracy of the expected kinetic parameters obtained from reaction progress curves analyses 25](#_Toc95732086)

[Appendix 2: MATLAB code to calculate the flux capacity and cofactor preference of AAR^Cn1^ and Chimera 5 28](#_Toc95732087)

# Supplementary Material 1: PHB generation pathway.


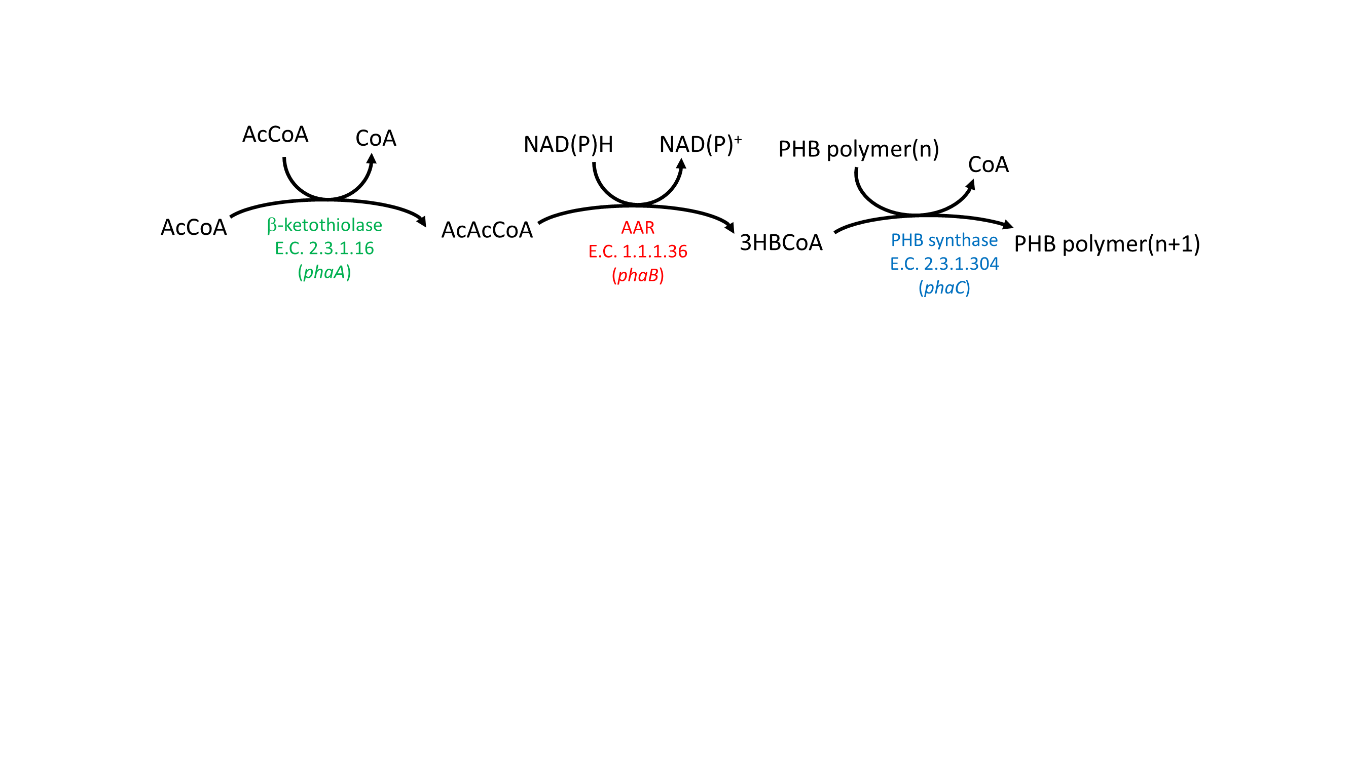


**Figure S1**: Two molecules of acetyl-CoA are condensed to form acetoacetyl-CoA (AcAcCoA), in the reaction catalyzed by a β-ketothiolase. Next, AcAcCoA is reduced to (R)3-hydroxybutyryl-CoA (3HBCoA) using NAD(P)H as the electron donor, in a reaction catalyzed by an acetoacetyl-CoA reductase (AAR). At last, a PHB synthase catalyzes the reaction of incorporation of one monomer of hydroxybutyrate into the existing polyhydroxybutyrate (PHB) polymer. The enzyme code numbers and the encoding genes (in parentheses) are indicated. In the case of *C. necator*, there are different paralog genes. The paralog genes placed in the operon *phaCAB1* are the most studied.

# Supplementary Material 2: DNA manipulations and protein purification

All DNA quantifications were performed using the Qubit 4 Fluorometer (Invitrogen), following the instructions of the manufacturer. PCRs were performed in a Biometra TAdvanced thermocycler (Westburg), using the protocol recommended by the DNA polymerase manufacturer. The *E. coli* strain DH5α (F-, Δ(*argF*-*lac*)169, φ80d*lacZ*58(M15), Δ*phoA8*, *glnX44*(AS), λ-, *deoR481*, *rfbC1*?, *gyrA96*(*NalR*), *recA1*, *endA1*, *thiE1*, *hsdR17*) was our choice for plasmid propagation and storage. On the other hand, the *E. coli* strain BL21(DE3) (fhuA2 [lon] ompT gal (λ DE3) [dcm] ∆hsdS λDE3=λsBamHIo ∆EcoRI-B int::(lacI::PlacUV5::T7 gene1) i21 ∆nin5) was our choice for the over-expression of recombinant proteins. Cellular growth of either DH5α or BL21(DE3) cells were performed in Lysogenic Broth (10 g Tryptone, 5 g yeast extract and 50 g NaCl for every 1 L distilled water, pH 7.0), with (15 g/L) or without agar.

Artificial DNA sequences encoding for the different acetoacetyl-CoA reductases under study were purchased at Integrated DNA Technologies (IDT, <https://eu.idtdna.com>). The DNA sequences of the ordered molecules are shown below:

>Chimera2

ATGACTCAGCGCATTGCGTATGTGACCGGCGGCATGGGTGGTATCGGAACCGCCATTTGCCAGCGGCTGGCCAAGGATG

GCTTTCGTGTGGTGGCCGGATGTGGTCCTGAATTTGATAAAGGAGAAAAGTGGTTAGAGCAGCAGAAGGCCCTGGGCTTC

GATTTCATTGCCTCGGAAGGCAATGTGGCTGACTGGGACTCGACCAAGACCGCATTCGACAAGGTCAAGTCCGAGGTCGG

CGAGGTTGATGTGCTGATCAACAACGCCGGTATCACCCGCGACGTGGTGTTCCGCAAGATGACCCGCGCCGACTGGGATG

CGGTGATCGACACCAACCTGACCTCGCTGTTCAACGTCACCAAGCAGGTGATCGACGGCATGGCCGACCGTGGCTGGGGC

CGCATCGTCAACATCTCGTCGGTGAACGGGCAGAAGGGCCAGTTCGGCCAGACCAACTACTCCACCGCCAAGGCCGGCCT

GCATGGCTTCACCATGGCACTGGCGCAGGAAGTGGCGACCAAGGGCGTGACCGTCAACACGGTCTCTCCGGGCTATATCG

CCACCGACATGGTCAAGGCGATCCGCCAGGACGTGCTCGACAAGATCGTCGCGACGATCCCGGTCAAGCGCCTGGGCCTG

CCGGAAGAGATCGCCTCGATCTGCGCCTGGTTGTCGTCGGAGGAGTCCGGTTTCTCGACCGGCGCCGACTTCTCGCTCAA

CGGCGGCCTGCATATGGGCTGA

>Chimera3

ATGACTCAGCGCATTGCGTATGTGACCGGCGGCATGGGTGGTATCGGAACCGCCATTTGCCAGCGGCTGGCCAAGGATG

GCTTTCGTGTGGTAGCAGGGTGCGGCCCCGAAGCGGACAAACCTGAAAAATGGCTTGAGCAACAGAAGGCCCTGGGCTTC

GATTTCATTGCCTCGGAAGGCAATGTGGCTGACTGGGACTCGACCAAGACCGCATTCGACAAGGTCAAGTCCGAGGTCGG

CGAGGTTGATGTGCTGATCAACAACGCCGGTATCACCCGCGACGTGGTGTTCCGCAAGATGACCCGCGCCGACTGGGATG

CGGTGATCGACACCAACCTGACCTCGCTGTTCAACGTCACCAAGCAGGTGATCGACGGCATGGCCGACCGTGGCTGGGGC

CGCATCGTCAACATCTCGTCGGTGAACGGGCAGAAGGGCCAGTTCGGCCAGACCAACTACTCCACCGCCAAGGCCGGCCT

GCATGGCTTCACCATGGCACTGGCGCAGGAAGTGGCGACCAAGGGCGTGACCGTCAACACGGTCTCTCCGGGCTATATCG

CCACCGACATGGTCAAGGCGATCCGCCAGGACGTGCTCGACAAGATCGTCGCGACGATCCCGGTCAAGCGCCTGGGCCTG

CCGGAAGAGATCGCCTCGATCTGCGCCTGGTTGTCGTCGGAGGAGTCCGGTTTCTCGACCGGCGCCGACTTCTCGCTCAA

CGGCGGCCTGCATATGGGCTGA

>Chimera4

ATGACTCAGCGCATTGCGTATGTGACCGGCGGCATGGGTGGTATCGGAACCGCCATTTGCCAGCGGCTGGCCAAGGATG

GCTTTCGTGTGGTTGCTGGGTGCGGGCCTGAGGAGGACCAACCAGAAAAGTGGTTGGAACAGCAGAAGGCCCTGGGCTTC

GATTTCATTGCCTCGGAAGGCAATGTGGCTGACTGGGACTCGACCAAGACCGCATTCGACAAGGTCAAGTCCGAGGTCGG

CGAGGTTGATGTGCTGATCAACAACGCCGGTATCACCCGCGACGTGGTGTTCCGCAAGATGACCCGCGCCGACTGGGATG

CGGTGATCGACACCAACCTGACCTCGCTGTTCAACGTCACCAAGCAGGTGATCGACGGCATGGCCGACCGTGGCTGGGGC

CGCATCGTCAACATCTCGTCGGTGAACGGGCAGAAGGGCCAGTTCGGCCAGACCAACTACTCCACCGCCAAGGCCGGCCT

GCATGGCTTCACCATGGCACTGGCGCAGGAAGTGGCGACCAAGGGCGTGACCGTCAACACGGTCTCTCCGGGCTATATCG

CCACCGACATGGTCAAGGCGATCCGCCAGGACGTGCTCGACAAGATCGTCGCGACGATCCCGGTCAAGCGCCTGGGCCTG

CCGGAAGAGATCGCCTCGATCTGCGCCTGGTTGTCGTCGGAGGAGTCCGGTTTCTCGACCGGCGCCGACTTCTCGCTCAA

CGGCGGCCTGCATATGGGCTGA

>Chimera5

ATGACTCAGCGCATTGCGTATGTGACCGGCGGCATGGGTGGTATCGGAACCGCCATTTGCCAGCGGCTGGCCAAGGATG

GCTTTCGTGTGGTTGCGGCTTATCATCCCGAGTTCGATAAGCCCGCCGAATGGACTAAACAACAGAAGGCCCTGGGCTTC

GATTTCATTGCCTCGGAAGGCAATGTGGCTGACTGGGACTCGACCAAGACCGCATTCGACAAGGTCAAGTCCGAGGTCGG

CGAGGTTGATGTGCTGATCAACAACGCCGGTATCACCCGCGACGTGGTGTTCCGCAAGATGACCCGCGCCGACTGGGATG

CGGTGATCGACACCAACCTGACCTCGCTGTTCAACGTCACCAAGCAGGTGATCGACGGCATGGCCGACCGTGGCTGGGGC

CGCATCGTCAACATCTCGTCGGTGAACGGGCAGAAGGGCCAGTTCGGCCAGACCAACTACTCCACCGCCAAGGCCGGCCT

GCATGGCTTCACCATGGCACTGGCGCAGGAAGTGGCGACCAAGGGCGTGACCGTCAACACGGTCTCTCCGGGCTATATCG

CCACCGACATGGTCAAGGCGATCCGCCAGGACGTGCTCGACAAGATCGTCGCGACGATCCCGGTCAAGCGCCTGGGCCTG

CCGGAAGAGATCGCCTCGATCTGCGCCTGGTTGTCGTCGGAGGAGTCCGGTTTCTCGACCGGCGCCGACTTCTCGCTCAA

CGGCGGCCTGCATATGGGCTGA

The received artificial DNA samples (approximately 500 ng) were dissolved in 50 µl of sterile DNA-free water and amplified by PCR. Aiming a very high-fidelity amplification, Q5 High-Fidelity DNA polymerase (New England Biolabs) was used. DNA samples were amplified using the primers p127 (5’-CTTATTGGATCCGATGACTCAGCGCATTGCGTATG-3’) and p128 (5’ GTGAATAAGCTTTCAGCCCATATGCAGGCCGCCGTTG-3’), which added to the template sequences (underlined) target sites for the restriction enzymes *Bam*HI and *Hind*III, respectively. Cleaned samples of the PCR products as well as a sample of the plasmid pCola-Duet1 (Novagen, Millipore) were digested (37 °C) using the restriction enzymes *BamHI* and *HindIII* (New England Biolabs), following the instructions of the enzyme manufacturer. To minimize events of self-ligation of the vector during the next step (ligation), after 1 hour and 45 minutes of digestion, calf intestinal alkaline phosphatase was added to the digestion mix containing the vector, followed by an additional incubation of 15 minutes (37 °C). Ligation was performed with T4 DNA ligase (New England Biolabs), using an insert:vector molar ratio of 3:1, for one hour (25 °C). Following ligation, cells of *E. coli* DH5α were transformed, by a heat shock (42 °C) , with the ligation products. Transformed cells were selected on plates filled with lysogenic broth agar supplemented with kanamycin (50 μg/ml). Plasmid purification was performed using the Monarch plasmid miniprep kit (New England Biolabs).

The presence of recombinant plasmid was first identified by colony PCR (One*Taq* DNA polymerase, New England Biolabs). Amplification patterns consistent with the expected constructions were observed in at least two colonies for each mutant. One colony per construction was arbitrarily picked for further plasmid isolation, sequencing (Baseclear, Leiden, NL) and preservation. After verification of the accuracy of the DNA constructions, the recombinant plasmids were introduced in *E. coli* BL21(DE3) cells (New England Biolabs), using the same heat shock protocol. The DNA sequences encoding for the mutant acetoacetyl-CoA reductases were ligated in the expression vector pCola-duet1 such that the over-expressed proteins have a 6x-poly-histidine tag in their N-terms. The DNA sequence maps (in GenBank format) of each one of the obtained plasmids are available in the repository Figshare database (figshare.com), under D.O.I. [10.6084/m9.figshare.16613794](https://doi.org/10.6084/m9.figshare.16613794)

*Protein purification*

For overexpression of the mutant acetoacetyl-CoA reductases, *E. coli* BL21(DE3) cells bearing the constructed plasmids were aerobically grown in Lysogenic Broth complemented with kanamycin, at 37°C. When the optical densities (600 nm) of the cultures approached values between 0.5 and 1.0, IPTG (Isopropyl β-D-1-thiogalactopyranoside) was added to the cultures up to a final concentration of 300 μM. Upon the addition of IPTG, incubation temperature was decreased to 25 °C and cells were harvested 12-16 hours later by centrifugation (1 hour, 16000x g, 4°C) (Sorvall RC 6 Plus Centrifuge, Thermo Scientific). Pellets were re-suspended in 20-30 mL of washing buffer (W buffer: Tris-HCl 50 mM, NaCl 5 mM, MgCl_2_ 5 mM, pH 8.0), centrifuged again (20 minutes, 2300x g, 4°C), and then the washed pellets were preserved at -20 °C until proceeding with the protein purification.

Protein purification started re-suspending the cellular pellets in equilibration buffer (EQ buffer: Tris-HCl 50 mM, NaCl 500 mM, MgCl_2_ 5 mM, imidazole 20 mM, pH 8.0) spiked with DL-dithiothreitol 2 mM (Sigma Aldrich) and EDTA-free protease inhibitor cocktail (cOmplet mini, Roche), using one tablet per 50 ml of EQ buffer. Cells were disrupted at 4°C using a CF1 Cell Disrupter (Constant Systems). The resultant suspension was centrifuged (1 hour, 45000x g, 4°C). Pellets were discarded and supernatants were kept ice-cold before the application in previously equilibrated His-trap columns (HisTrap FF 5 ml, GE Healthcare), following the instructions of the manufacturer. Equilibration of the columns was performed following the instructions of the manufacturer and the flow rate was set to 5 mL/min. After column equilibration, supernatants carrying the cytoplasmic proteins were loaded onto the columns. After loading the columns, washings with EQ buffer were performed aiming to remove the unbound proteins and cellular debris from the columns. Washings with EQ buffer were performed until the buffer leaving the columns does not carry a concentration of proteins detectable using the standard Quick Protein Assay (Bio-Rad). Protein elution was accomplished by increasing the concentration of imidazole in the buffer going into the columns. A concentration gradient was setup using the EQ buffer and the elution buffer (EB buffer, Tris-HCl 50 mM, NaCl 500 mM, MgCl_2_ 5 mM and imidazole 500 mM, pH 8.0). The protein concentration in the collected fractions was determined using the Quick Protein Assay (Bio-Rad) and the obtained values were plotted. Fractions with protein concentration values in the upper quartile were pooled and placed in a dialysis bag. Sequential dialysis steps were performed aiming (i) the removal of imidazole, (ii) a gradual decrease in NaCl concentration up to 5 mM, keeping Mg^2+^ concentration in 5 mM and (iii) a change in pH from 8.0 (more suitable for IMAC-based protein purification) to 7.0 (required for substrates stability during the kinetic assays). The first dialysis was against W buffer. After two hours of dialysis, W buffer was gradually substituted by a transition buffer ((3-(N-morpholino)-propanesulfonic acid, MOPS) 50 mM, NaCl 5 mM, MgCl_2_ 5 mM, pH 7.0, 10 % (v/v) glycerol, glutamic acid 25 mM, arginine 25 mM). Finally, the transition buffer surrounding the dialysis bag was gradually substituted by MOPS buffer (MOPS 50 mM, NaCl 5 mM, MgCl_2_ 5 mM, pH 7.0). Purity and molecular size of the final protein samples were checked by SDS-PAGE chromatography (see **Supplementary Material 3**).

To prevent eventual inactivation during the storage at -20 °C, glycerol 99 % with quality suitable for molecular biology procedures (Sigma product code G5516) was gradually added to the dilutions containing the purified proteins such that the final partition between protein dilution and glycerol was 50:50 %. The enzymatic assays were performed with enzyme preparations stored at -20 °C for no more than 48 hours.

# Supplementary Material 3: Quantification of the reagents

After finishing the protein purification procedures, purity and molecular weight of the enzymatic samples were assessed by SDS-PAGE chromatography (**Figure S2**). To determine the protein concentration in the enzymatic dilutions, two approaches were compared: (i) estimation based on measuring directly the absorbance at 280 nm of the enzyme dilutions, and (ii) estimation based on the method developed by Bradford ^1^, using the Bio-Rad protein assay (product code 5000001). In both cases, MOPS buffer ((3-(N-morpholino)-propanesulfonic acid) 50 mM, NaCl 5 mM, MgCl_2_ 5 mM, pH 7.0) was used as the blank solution.


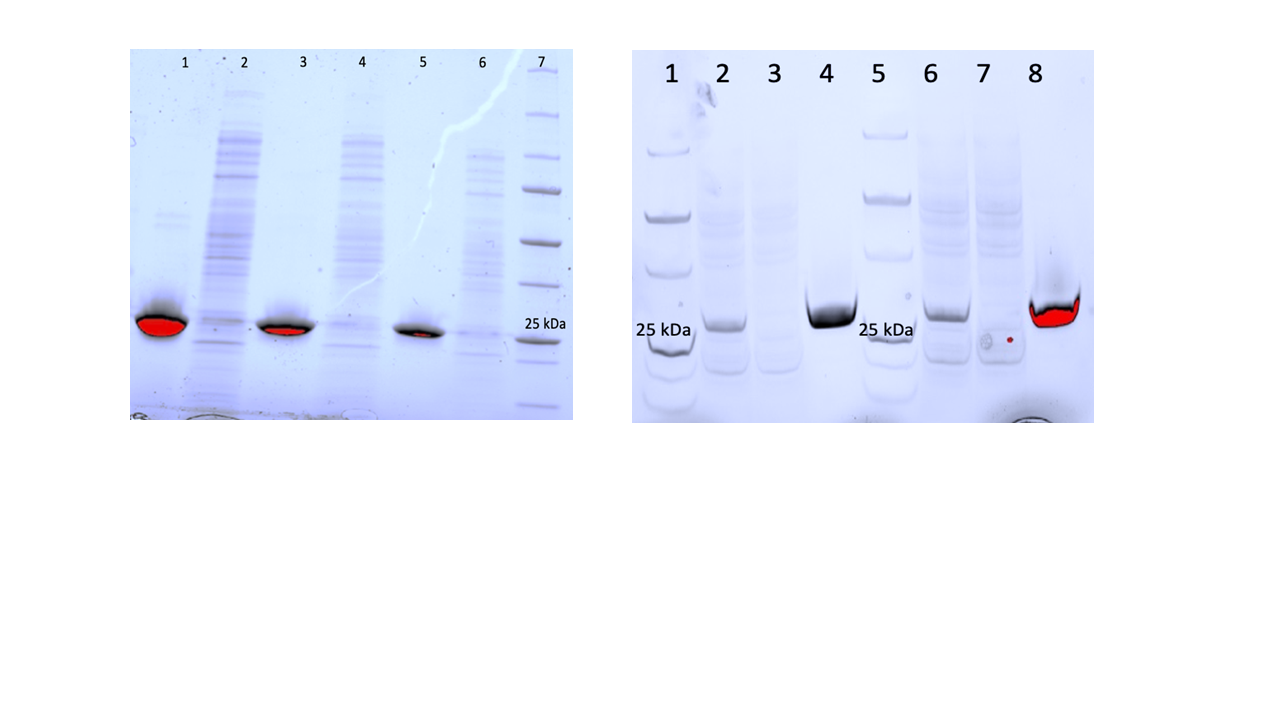


**Figure S2**: SDS-PAGE chromatography to verify the purity and molecular weight of the enzymes. In the left side, it is possible to see different amounts of AAR^Cn1^ (lanes 1, 3 and 5 were loaded with 20 μg, 10 μg and 5 μg respectively), and comparable amounts (lanes 2, 4 and 6) of the cell-free extract obtained from the BL21(DE3) cells transformed with the plasmid pCola-phaBCnecator, collected after 12 hours of induction with IPTG. In the right side, it is possible to see samples of cell-free extracts (lanes 2 and 6) obtained from the BL21(DE3) cells transformed with the plasmid pCola-Chimera5, collected after 16 hours of induction with IPTG; cell-free extracts (lanes 3 and 7) after flowing through the nickel-charged column (HisTrap FF 5 ml, GE Healthcare); and samples of the purified protein Chimera 5 (lanes 4 and 8). Lanes 2 to 4 contain 4 μg of protein, and lanes 6 to 8 contain 8 μg of protein. This latter photo enables to see the effectiveness of the nickel-charged column retaining the poly-histidine tagged proteins. Lane 7 (photo in the left side), and lanes 1 and 5 (photo in the right side) were loaded with a sample of a molecular mass ruler (Precision Plus Protein™ Unstained Standards, Bio-Rad). The standard with a size closest to the expected size of the purified protein (~28 kDa) is labelled in the photo. Using the loading dye 4x Laemmli Sample Buffer (Bio-Rad), no staining of the gel was required, as the versions of the acetoacetyl-CoA reductase under study contain a tryptophan percentage higher than 1.5 %.

For the first method, extinction coefficients were calculated from the amino acid sequences of the enzymes using the ProtParam tool (https://web.expasy.org/protparam/) ^2^. The obtained values were 32095 M^-1^cm^-1^ (assuming all pairs of Cys residues form cystines) and 31970 M^-1^cm^-1^ (assuming all Cys residues are reduced). Regarding the protein concentration estimations using the Bio-Rad protein assay, dilutions of Bovine Serum Albumin in MOPS buffer, at known concentrations, were used as standards. Each protein concentration assessment was performed five independent times, with a coefficient of variation lower than 5 %. Enzyme concentrations estimated using the Bio-Rad protein assay were consistently 1.3 higher than the estimates obtained from the direct absorbance at 280 nm (M_w_ = 27948.58 Da; ε = 31970 M^-1^cm^-1^). Given the unknown status of the cysteine residues in the purified proteins and the simplicity of the procedure, we chose to determine the enzyme concentrations for the definitive experiments using the Bio-Rad protein assay. The stability of the enzyme during the reactions was evaluated using the test developed by Selwyn ^3^. According to our observations, enzyme concentrations in the reactions above 0.5 nM were considered stable in the conditions on the assay (**Figure S3**).

On the other hand, AcAcCoA stocks were prepared in distilled water (slightly acid pH favors the stability of AcAcCoA solutions). However, the substrate concentrations in these freshly prepared AcAcCoA stocks were assessed as follows: a solution (at a known dilution) of the freshly prepared stock was prepared in W buffer. The absorbance of the diluted solution, at 310 nm, was recorded, using W buffer as the blank. In these conditions (pH 8.0 and Mg^2+^ 5 mM), the molar extinction coefficient ε^310nm^ = 11000 M^-1^cm^-1^ ^4^ was considered for the calculation of the concentration in the freshly prepared AcAcCoA stock. The use of W buffer ensured stable and known H^+^ and Mg^2+^ concentrations, an important question considering that the molar extinction coefficient of AcAcCoA steeply change with small changes in Mg^2+^ and H^+^ concentrations, as carefully studied by Stern ^4^. In the case of NAD(P)H, the stocks were prepared and quantified in MOPS buffer. The reference molar extinction coefficient of ε^340nm^ = 6220 M^-1^cm^-1^ was employed to evaluate the concentrations of the NAD(P)H stocks. To determine the apparent extinction coefficients to be used outside the reference wavelengths, dilutions of AcAcCoA and NAD(P)H in MOPS buffer, were prepared and their absorbance spectrum from 310 nm to 360 nm were measured. Upon calculation of the concentrations of the stocks using the absorbance recorded at the reference wavelength, the apparent extinction coefficients outside the reference wavelength were calculated using the recorded absorbance and the calculated concentrations. The obtained apparent extinction coefficients are: ε_AcAcCoA_^340nm^ = 45 M^-1^cm^-1^, ε_AcAcCoA_^360nm^ = 23 M^-1^cm^-1^ and ε_NAD(P)H_^360nm^ = 4075 M^-1^cm^-1^.

The stability of the NADH and NADPH solutions in MOPS buffer, at 30°C, was assessed observing the temporal changes in their absorbance at 340 nm. The absorbance data were the input to calculate the changes in concentrations in time. The observed temporal changes were adjusted to a first-order decay process. The first-order decay constants (τ) were 0.434 x 10^-5^ s^-1^ for NADH and 1.28 x 10^-5^ s^-1^ for NADPH. Taking the τ = 1.28 x 10^-5^ s^-1^ observed for NADPH as the worst case scenario, this value means that in two hours less than 10% of the NADPH is degraded. Thus, the effect of NAD(P)H degradation, during the short term incubations on ice or during the reactions, was neglected. However, NAD(P)H solutions were always freshly prepared and their concentrations were evaluated before each experimental session. Regarding the AcAcCoA solutions, they showed a stable absorbance at 310 nm, during at least 2 hours, when placed on ice.

Finally, the potential effects of water evaporation were also evaluated. Using 96 wells half-area microplates (Greiner, code 675101), an initial volume of 80 µl of MOPS buffer was reduced to 54 µl after two hours of incubation at 30°C inside the plate reader (Synergy HTX, Biotek). Thus, water evaporation rate was 0.21 µl/min. This means a variation in the volume of less than 8 % in 30 minutes. Therefore, in our kinetic analyses, we did not include the effects of water evaporation on substrates, products and enzyme concentrations.

**Figure S3**: Selwyn plot obtained with different concentrations of AAR^Cn1^. The initial concentrations of AcAcCoA and NADPH were 100 µM, and the reaction was studied in MOPS buffer at 30 °C. The reactions were setup in 96 wells half-area microplates (Greiner, code 675101), with an initial volume of 100 µl. Evidence of inactivation was detected only for the enzyme concentration 0.25 nM (curve corresponding to this enzyme concentration does not overlap the other curves).

# Supplementary Material 4: Quantification of product concentration during the enzymatic assays

Under our experimental conditions, any recorded absorbance *Abs_i_* is the result of the following sum:

$${Abs}_{i}={Abs}_{blank}+c_{i}^{NAD\left( P \right)H}*\varepsilon^{NAD\left( P \right)H}*l+c_{i}^{AcAcCoA}*\varepsilon^{AcAcCoA}*l$$

where *Abs_blank_* is the average absorbance of 12 wells filled with MOPS buffer (using the reaction volume), *l* is the optical path length, *ε^NADH^* is the molar extinction coefficient of NAD(P)H and *ε^AcAcCoA^* is the extinction coefficient of AcAcCoA. The next recorded absorbance *Abs_i+1_* is the result of the following sum:

$${Abs}_{i+1}={Abs}_{blank}+c_{i+1}^{NAD\left( P \right)H}*\varepsilon^{NAD\left( P \right)H}*l+c_{i+1}^{AcAcCoA}*\varepsilon^{AcAcCoA}*l$$

Thus, the variation in the absorbance between these two records (*ΔAbs*) is:

$$\Delta Abs={\Delta c}^{NAD\left( P \right)H}*\varepsilon^{NAD\left( P \right)H}*l+{\Delta c}^{AcAcCoA}*\varepsilon^{AcAcCoA}*l$$

where *Δc^NAD(P)H^* is the variation in NAD(P)H concentration and *Δc^AcAcCoA^* is the variation in AcAcCoA concentration. But, given the stoichiometry of the reaction, the variation in the product concentration between these two records is:

*Δc^NAD(P)H^* = *Δc^AcAcCoA^* = (*ΔP*) = *P_i+1_* - *P_i_*.

Therefore:

$$\left( \Delta P \right)= P_{i+1} - P_{i}= \frac{\left| {Abs}_{i}-{Abs}_{i+1} \right|}{l*(\varepsilon^{NADH}+\varepsilon^{AcAcCoA})}$$

or more generally,

$$P\left( t \right)=\frac{{Abs}_{mix}^{expected}-{Abs}_{t}}{l*(\varepsilon^{NADH}+\varepsilon^{AcAcCoA})}$$

where ${Abs}_{mix}^{expected}$ is the expected absorbance when the reaction mix was initially assembled. This expected initial absorbance was calculated as:

$${Abs}_{mix}^{expected}={Abs}_{blank}+c_{0}^{NAD\left( P \right)H}*\varepsilon^{NAD\left( P \right)H}*l+c_{0}^{AcAcCoA}*\varepsilon^{AcAcCoA}*l$$

where $c_{0}^{NAD\left( P \right)H}$ and $c_{0}^{AcAcCoA}$ are the concentrations of NAD(P)H and AcAcCoA in the initial reaction mix.

We also calculated the actual substrate (*S_0_*) and product (*P_0_*) concentrations at the beginning of each experimental record as:

$$S_{0}=c_{0}^{NAD\left( P \right)H or AcAcCoA}-P_{0}$$

where *P_0_* is the product formed during the lag time (the time lapse between the mixing of the reagents and the record of the first absorbance in time):

$$P_{0}=\frac{({Abs}_{mix}^{expected}-{Abs}_{b})}{l*(\varepsilon^{NADH}+\varepsilon^{AcAcCoA})}$$

where *Abs_b_* is the basal absorbance (operationally calculated as the minimal absorbance recorded during the first minute). For a more accurate description of the reactions, during the parameter estimations we considered the calculated *S_0_* and *P_0_* values instead of assuming *S_0_* = $c_{0}^{NAD\left( P \right)H or AcAcCoA}$ and *P_0_* = 0.

# Supplementary Material 5: A simulation tool to evaluate the accuracy of the kinetic parameters assessed by reaction progress curves analysis

*Initial rates vs reaction progress curves*

Enzyme kinetic parameters can be estimated measuring the **initial rates** at different concentrations of the substrate(s) or through **reaction progress curves** analysis ^5,6^. The mathematical treatment to obtain the kinetic parameters from initial rates data is simpler than the reaction progress curves analysis ^7^. However, to obtain estimations of the kinetic parameters with a narrow dispersion using the initial rates approach it is often necessary to measure the reaction rates at substrate concentrations around or above K_M_ ^8^, and the amount of substrate consumed during measurement of the initial rates should be below 1 % ^5^. These conditions are not always experimentally feasible. In those cases, the analysis of reaction progress curves could be a solution.

*The use of the Michaelis-Menten model to study BiBi reactions*

On the other hand, the majority of the reactions catalyzed by enzymes have two substrates ^5^. In these cases, for the sake of simplicity, it is convenient to observe, separately, the effect of changing the initial concentration of one of the substrates while keeping fixed and saturating the initial concentration of the other substrate. Under such conditions, it is often assumed that the kinetic behavior of the system can be described as an irreversible, mono-substrate and mono-product reaction (simple Michaelis-Menten model) ^6^. For example, keeping the substrate *B* at a fixed concentration while the second substrate *A* is varied, and considering that product concentrations are low at the early stages of the reactions, it is possible to determine the kinetic parameters *k_cat_* and *K_MA_* from the analysis of the **initial rates** (*v_0_*) ^5,6^:

$$\frac{initial variation in the product concentration}{time}=v_{0}=\frac{E*k_{cat}^{app}*A}{K_{MA}^{app}+A}$$

Notice that we added the super index *app* to *k_cat_* and K_M_ to indicate the parameters obtained under such conditions are “apparent”, *i.e.*, they depend on the chosen substrate *B* concentration. A similar experimental approach can be employed to determine the kinetic parameters through the **reaction progress curves** analysis:

$$\frac{dA}{dt}=\frac{E*k_{cat}*A(t)}{K_{MA}+A(t)}$$

Nevertheless, in the case of the reaction progress curves, the inhibitory effects of the accumulating products could have an important impact on the reaction rates. Moreover, depending on the proximity to the thermodynamic equilibrium, the reverse reaction could also have a major effect on the reaction rates. Therefore, *it is very important to choose the right experimental conditions where it is possible to use the simple Michaelis-Menten model to obtain the relevant kinetic parameters of reversible BiBi reactions using data coming from reaction progress curves*. In these cases, the accuracy of the parameters obtained using the Michaelis-Menten model will depend on how far from thermodynamic equilibrium the reactions are during the data recording, the catalytic power of the enzyme, the saturation constants, the data acquisition rate and the experimental errors. We developed a simulation tool to *in silico* evaluate the effects of all these factors on the accuracy of the kinetic parameters. Briefly, the procedure of the simulation tool is as follows.

First, different progress curves, corresponding to different initial concentrations of one of the substrates, are generated *in silico*. To generate the concentrations in time data for such reaction progress curves, it is necessary to integrate a differential equation describing how the concentration of one of the products change in time. The chosen differential equation depends on the expected reaction mechanism. For our simulation tool, we chose the full rapid-equilibrium mechanism because (i) among the known BiBi mechanisms, it has the smaller number of kinetic parameters and (ii) using this mechanism, it is possible to represent with fair accuracy the behavior of more complex mechanisms ^9^. For the reaction A+B = P+Q, we can write the differential equation describing the changes in the concentration of the product P in time as follows:

$$\frac{dP}{dt}= \frac{k_{cat}^{f}*E* \frac{A(t)*B(t)}{K_{A}* K_{B}}*\left( 1- \frac{P(t)*Q(t)}{A(t)*B(t)* K_{eq}} \right)}{\left( 1+ \frac{A(t)}{K_{A}}+ \frac{Q(t)}{K_{Q}} \right)*\left( 1+ \frac{B(t)}{K_{B}}+ \frac{P(t)}{K_{P}} \right)}$$

where *k*^f^*_cat_* is the turnover constant in the forward direction, K_eq_ is the thermodynamic equilibrium constant, and K_A_, K_B,_ K_P_ and K_Q_ are the dissociation constants characterizing the interactions of the ligands A, B, P and Q with any form of the enzyme (**Figure S4A**). *E* stands for enzyme concentration, which typically are two to four orders of magnitude less concentrated than substrates. Following simple stoichiometric rules, the changes in the concentration of A, B and Q are described with the equations A(t) = A(t_0_) - P(t); B(t) = B(t_0_) - P(t); Q(t) = Q(t_0_) + P(t).

To simulate random experimental errors, pseudo-random normally distributed error (5 %) was artificially added to the simulated reaction progress curves. After adding this noise, the records of P(t) were used as the input to obtain the kinetic parameters through the fitting to the analytical solution of the integrated form of the Michaelis-Menten equation ^10^:

$$P\left( t \right)=A_{o}- K_{MA}*W\left( \frac{A_{o}}{K_{MA}}e^{\frac{-k_{cat}^{f}*E*t+ A_{o}}{K_{MA}}} \right)$$

where *W* is the omega function, which satisfies the transcendental equation: $W\left( x \right)*e^{W\left( x \right)}=x$.

A visual representation of the described procedure is shown in **Figure S4**. The simulation tool was written in MATLAB, and its code, with commentaries, is provided at the end of these supplementary materials.

*The specific case of the acetoacetyl-CoA reductases*

The reactions catalyzed by acetoacetyl-CoA reductases can be classified as reversible BiBi. One way to achieve experimental conditions where the simple Michaelis-Menten model could be applied is through the consumption of at least one of the products in a coupled downstream reaction. Nevertheless, the most common reactions consuming 3-hydroxybutyryl-CoA or NAD(P)^+^ generate other products that can interfere with the reaction catalyzed by the acetoacetyl-CoA reductase. Then, the standardization of an enzymatic assay to measure the acetoacetyl-CoA reductase activity, including a coupled downstream reaction removing one of the products, is not straightforward. Therefore, we decided to first measure some kinetic parameters using the conventional initial rates approach, without any coupled reaction.

Using a sample of purified AAR^Cn1^, we monitored the early stages of reactions starting with different concentrations of NADH or NADPH. In the assays using NADPH as cofactor, the initial AcAcCoA concentration was 200 µM (K_M_^AcAcCoA^ values of 5 µM and 5.7 µM has been reported for acetoacetyl-CoA reductases from *C. necator*, using NADPH ^11,12^). In the assays using NADH as cofactor, the initial AcAcCoA concentration was 800 µM. No direct experimental estimations of K_M_^AcAcCoA^ values for AAR^Cn1^ using NADH has been reported. However, to the best of our knowledge, all the experimental values of K_M_^AcAcCoA^ so far reported are below 80 µM.

Preliminary estimations of *k_cat_* and K_M_^NAD(P)H^ were obtained by fitting the initial rates to the simple Michaelis-Menten model. In the case of the reactions using NADPH, the obtained parameters (*k_cat_* = 5 s^-1^, K_M_^NADPH^ = 24 µM) are similar to the estimates obtained by Haywood and co-workers (*k_cat_* = 15 s^-1^, K_M_^NADPH^ = 19 µM) ^11^. However, in the case of the reactions using NADH, the 95% confidence intervals included negative (physically meaningless) values (**Figure S5 bottom**). A similar situation was observed during the study of the initial rates of reactions catalyzed by three of the four engineered enzymes (**Figure S6**). Thus, we reasoned that it is possible that the observed poor fitting is the result of exploring the initial rates at NAD(P)H concentrations far below their respective K_M_ values. However, the increment in NAD(P)H concentrations could provoke the optical artifact known as stray light, leading to erroneous rate estimations ^13^. To overcome this problem, we decided to obtain the kinetic parameters from the analysis of the reaction progress curves. The previously described simulation tool was employed to explore the suitable substrate concentration ranges, enzyme concentration and recording time where the use of the simple Michaelis-Menten model yields estimates for the kinetic parameters with an acceptable accuracy.

Assigning K_A_ to K_M_^NADH^, K_B_ to K_M_^AcAcCoA^, K_P_ to K_M_^3HBCoA^ and K_Q_ to K_M_^NAD^, we simulated situations where acetoacetyl-CoA (AcAcCoA) was initially present at 2 mM (a concentration well-above all the reported K_M_^AcAcCoA^), and cofactor concentrations varying from 10 µM to 600 µM. The experimentally determined equilibrium constant K_eq_ = 92 ^14^ was employed for the simulations. Regarding the enzyme concentrations, we tested the values 1 nM and 4 nM. According to a previously performed Selwyn test, in our experimental conditions the purified enzymes are stable at concentrations above 0.5 nM (**Figure S3**).

According to the *in silico* simulations, using an AcAcCoA concentration ≥ 1.6 mM, NAD(P)H concentrations changing between 10 and 600 µM, enzyme concentrations ≤ 4 nM, and analyzing reaction time windows of up to 30 minutes, it should be possible to obtain kinetic parameters deviating less than 10 % from the expected values in all the explored cases, with the exception of the extreme situation of having an acetoacetyl-CoA reductase with a high *k_cat_* used at a high enzyme concentration during the enzymatic assay (**Figure S7**).


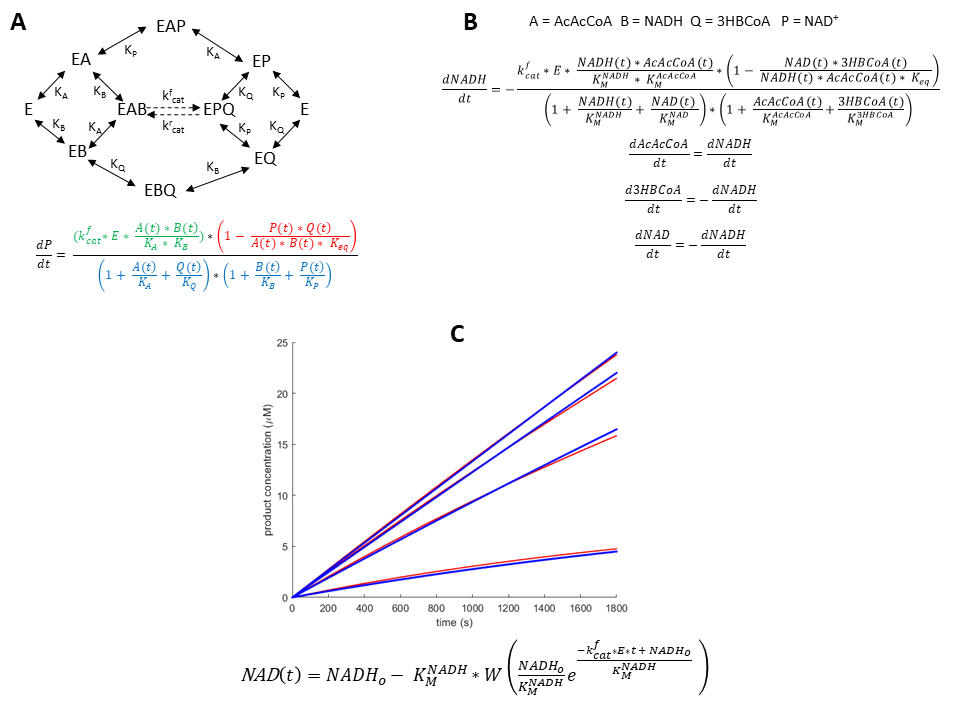


**Figure S4**: Visual explanation of the procedure to perform an i*n silico* comparison between the kinetic parameters of a reversible BiBi reaction and the kinetic parameters obtained upon the assumption that such reaction is an irreversible UniUni reaction. **A**: First, we assumed that the enzyme catalyzing the reversible BiBi reaction has a simplified full rapid-equilibrium mechanism. The rate equation for this case has been proposed by Rohwer and co-workers ^9^ and it is possible to identify in the equation one term expressing the catalytic power of the enzyme (in green), another term expressing how far from the thermodynamic equilibrium the reaction is (in red) and terms expressing the saturation state of the enzyme (in blue). **B**: It is possible to write a system of differential equations to calculate the expected variations in time for the substrates and products of the reaction catalyzed by the acetoacetyl-CoA reductases, under such mechanism. **C**: Product (NAD^+^) concentrations in time (generated with the system of differential equations represented in **B**), plus some added error, are the pseudo-experimental values employed as input to determine the best-fitted $k_{cat}^{f}$ and $K_{M}^{NADH}$, using the closed-form solution of the integrated form of the Michaelis-Menten equation ^10^. For simplicity, in the graphical representation are included only four pseudo-experimental progress curves (red dots) and the best-fitted solutions (blue lines) found with the closed form solution, for the time window of the first 30 minutes of the reaction.


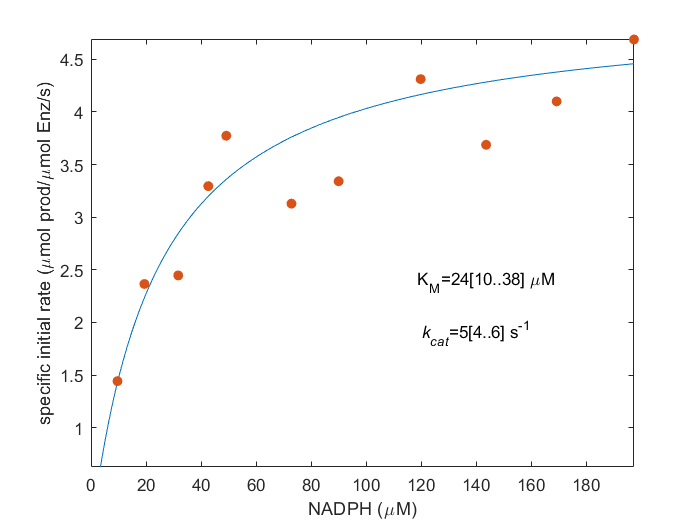


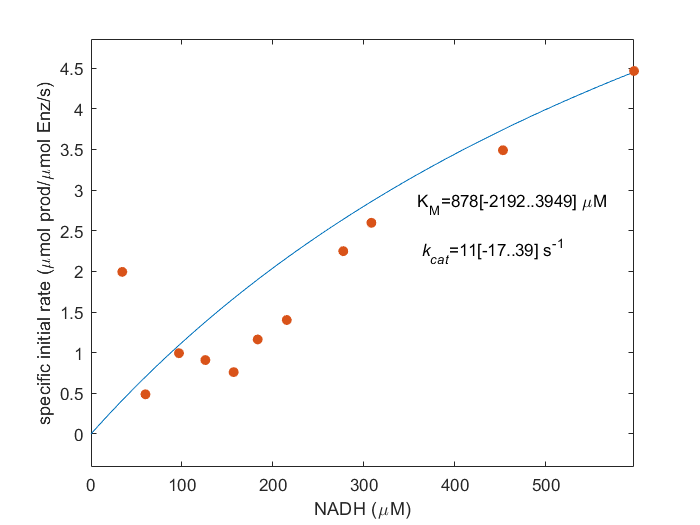


**Figure S5**: Initial rates of different reactions catalyzed by AAR^Cn^, using NADPH (top) or NADH (bottom) as cofactors. Kinetic parameters (inside the graphics, best fit value [95 % confidence intervals]) were obtained by fitting the initial rates to the equation (v_0_/E) = (*k_cat_**S)/(K_M_+S).


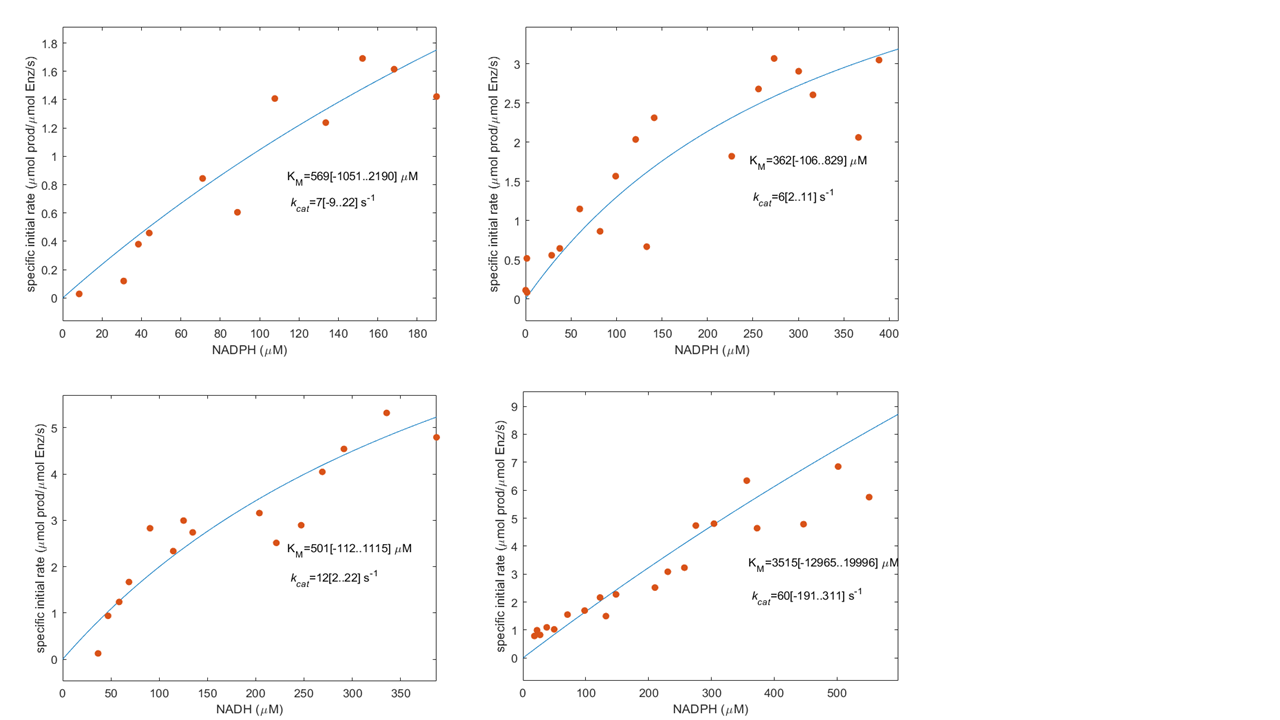


**Figure S6**: Examples of initial rates at different NAD(P)H concentrations poorly fitted to the simple Michaelis-Menten model. The graphs represent records from reactions catalyzed by Chimera 2 (top left), Chimera 4 (bottom left and top right) and Chimera 5 (bottom right). Inside the graphics are presented the kinetic parameters (best fit value [95 % confidence intervals]).

**
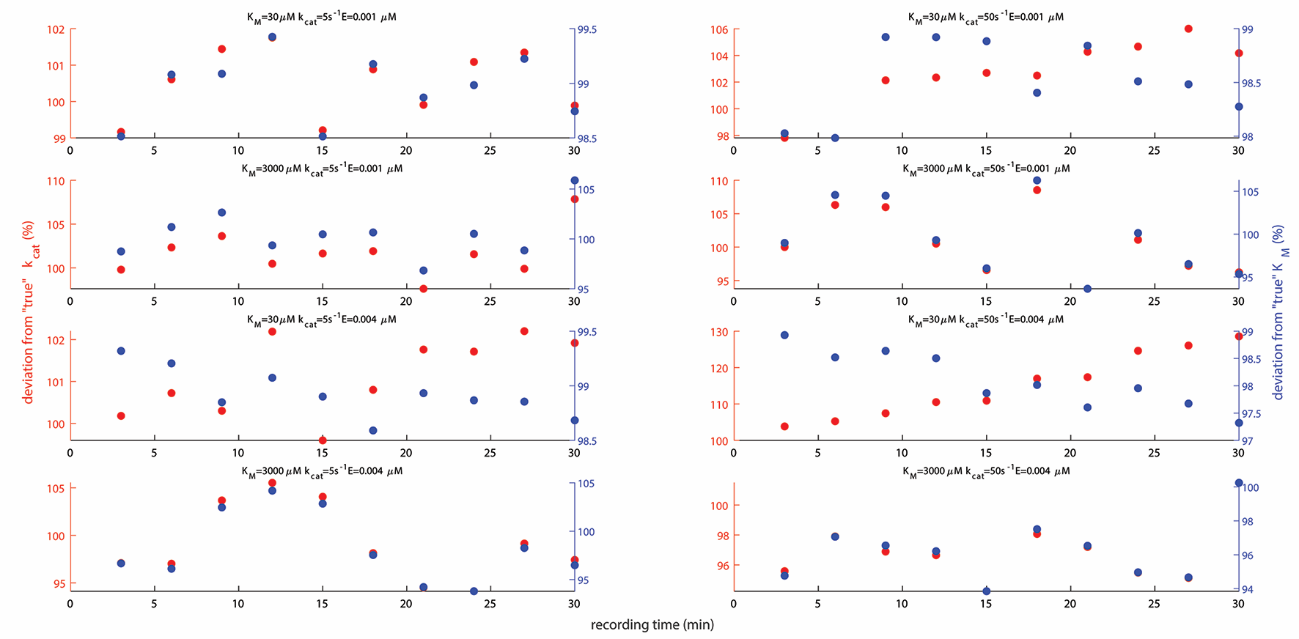
**

**Figure S7**: Accuracy of the statistical estimations of the parameters *k_cat_* and K_M_ when the integrated form of the Michaelis-Menten equation was employed to fit data from simulated progress curves of a reversible A + B = P + Q reaction. The reaction progress curves, considering different combinations of K_M_A, *k_cat_* and enzyme concentration, were simulated using the simplified full rapid-equilibrium enzymatic mechanism proposed by Rohwer and co-workers ^9^. Substrate B had an initial concentration of 2000 µM (20 times K_M_B), and the simulated concentrations of the substrate A varied between 10 and 600 µM. The individual “true” *k_cat_*, “true” K_M_A and enzyme concentrations used for these simulations are shown in each specific graph. Normally distributed 5 % noise was added to the simulated reaction progress curves before the execution of the statistical fitting procedure to obtain the *k_cat_* and K_M_A estimates. Different time windows (recording times, in minutes) along the progress curves were selected to fit these datasets with the closed-form solution of the integrated Michaelis-Menten equation. The best-fitted *k_cat_* (blue dots) and K_M_A (red dots) are represented as percentages of the “true” kinetic parameters.

# Supplementary Material 6: Biochemical mechanisms under consideration

The software DYNAFIT (Biokin) ^15^ can perform model discrimination analyses to verify whether a given (bio)chemical model can explain the kinetic patterns observed in a given set of (bio)chemical reactions. The steps of the mechanism of the (bio)chemical process under consideration must be suitable to be represented using the mass-action law. Reversible interactions are represented with the symbol <===> and the irreversible processes are represented with the symbol -->. The corresponding rate constants are declared after the colon (:). For example, the simple Michaelis-Menten model is written as follows:

E + S <===> ES : *k1 kminus1*

ES --> E + P : *kcat*

In this specific study, we did not determine the values of the individual rate constants behind the reversible dissociation processes (for example, we did not determine the individual values of *k1* and *kminus1*). We did determine the values of the kinetic parameters K_M_ and *k_cat_*. Under the rapid-equilibrium assumption, K_M_ = *kminus1*/*k1*. To solve the statistical problem having three unknowns (*k1*, *kminus1* and *k_cat_*) and only two not linearly dependent processes, we arbitrarily fixed the value *k1*=100. This way, K_M_ values were determined by dividing the best fitted values for *kminus1* over 100. Therefore, the individual values of *k1* and *kminus1* are conditioned by the arbitrary choice *k1*=100, but the ratio *kminus1*/*k1* is the best fitted estimate for K_M_.

Beyond the simple Michaelis-Menten model, for the reactions where NAD(P)H concentrations were changed while keeping constant the initial AcAcCoA concentration, we also evaluated the possibility of the following mechanisms:

**Competitive product inhibition**

E + S <===> ES : k1 kminus1

ES --> E + P : kcat

E + P <===> EP : k1 kp

**Noncompetitive product inhibition**

E + S <===> ES : k1 kminus1

ES --> E + P : kcat

ES + P <===> ESP : k1 kp

**Mixed product inhibition**

E + S <===> ES : k1 kminus1

ES --> E + P : kcat

ES + P <===> ESP : k1 kp

E + P <===> EP : k1 kp2

In the cases of reactions where AcAcCoA concentration was varied while keeping constant the initial NAD(P)H concentrations, we evaluated these other mechanisms:

**Typical Substrate Inhibition**

E + S <===> ES : k1 kminus1

ES --> E + P : kcat

ES + S <===> ES2 : k1 kminus2

**Special case of Substrate Inhibition with K_iS_=K_M_**

E + S <===> ES : k1 kminus1

ES --> E + P : kcat

ES + S <===> ES2 : k1 kminus1

**Substrate and product inhibition**

E + S <===> ES : k1 kminus1

ES --> E + P : kcat

ES + S <===> ES2 : k1 kminus2

E + P <===> EP : k1 kp

**Mixed inhibition with an inactive ESS complex**

E + S <===> ES : k1 kminus1

ES --> E + P : kcat

E + S <===> ESb : k1 Koff1

ES + S <===> ESS : k1 Koff2

# Supplementary Material 7: Relative use of NADH over NADPH at high AcAcCoA concentration

Given the lack of information about what is the mechanism of the reaction catalyzed by the acetoacetyl-CoA reductases under study, as a first approximation we could describe the reaction rates using the generic BiBi equation proposed by Rohwer and co-workers (see Supplementary Material 5 for details):

$$v= \frac{k_{cat}^{f}*E* \frac{NAD(P)H*AcAcCoA}{K_{AcAcCoA}* K_{NAD\left( P \right)H}}*\left( 1- \frac{NAD(P)*HBCoA}{AcAcCoA*NAD\left( P \right)H* K_{eq}} \right)}{\left( 1+ \frac{AcAcCoA}{K_{AcAcCoA}}+ \frac{HBCoA}{K_{HBCoA}} \right)*\left( 1+ \frac{NAD\left( P \right)H}{K_{NAD\left( P \right)H}}+ \frac{NAD(P)}{K_{NAD(P)}} \right)}$$

At the beginnings of the reaction [NAD(P)] = [HBCoA] ≈ 0. Thus, it is possible to write:

$$v= \frac{k_{cat}^{f}*E* NAD\left( P \right)H}{\left( K_{NAD\left( P \right)H}+ NAD\left( P \right)H \right)*\left( 1+ \frac{K_{AcAcCoA}}{AcAcCoA} \right)}$$

If [AcAcCoA] >> K_AcAcCoA_ (saturating AcAcCoA concentration), K_AcAcCoA_/AcAcCoA ≈ 0, it is possible to write:

$$v= \frac{k_{cat}^{f}*E* NAD\left( P \right)H}{\left( K_{NAD\left( P \right)H}+ NAD\left( P \right)H \right)}$$

which is mathematically equivalent to the simple Michaelis-Menten equation. For these specific conditions, it is possible to predict the specific rates (at unitary enzyme concentration) of the enzymes under study at different concentrations of NADH and NADPH (**Figure S8**).


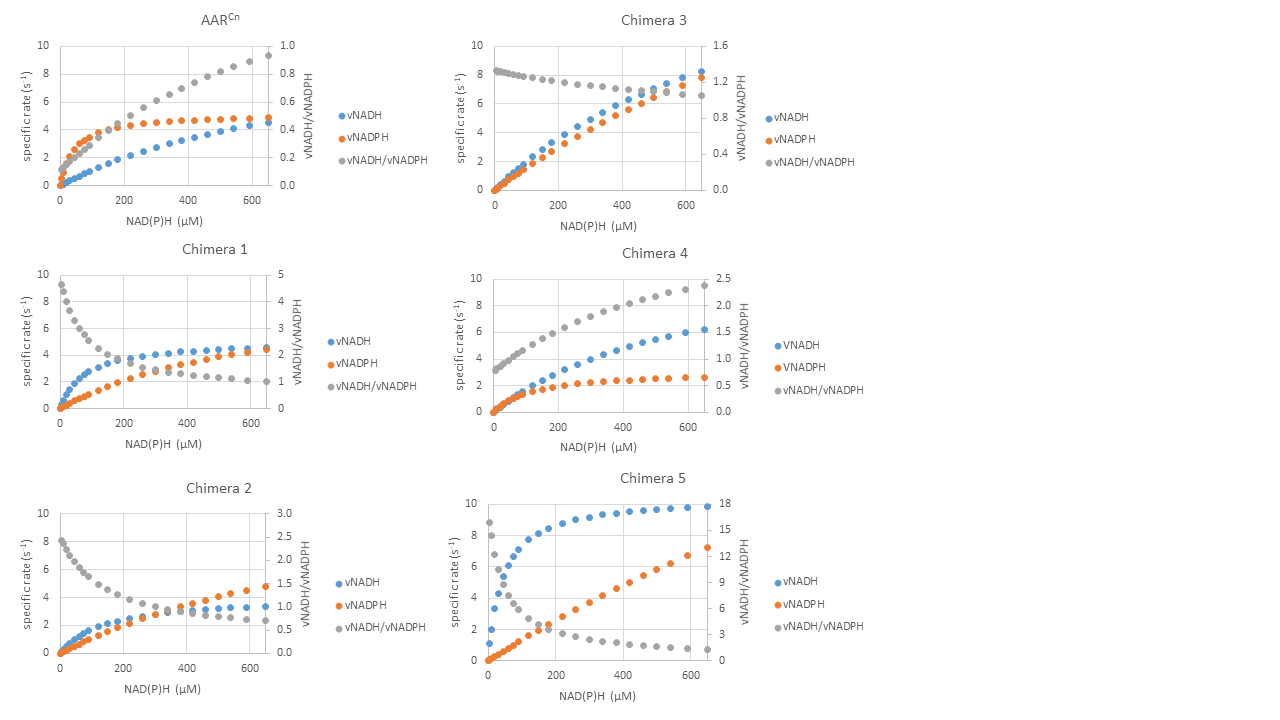


**Figure S8**: Specific activities of different acetoacetyl-CoA reductases, using NADH or NADPH, calculated using the Michaelis-Menten equation, assuming a unitary enzyme concentration and a saturating AcAcCoA concentration. In the right *y* axis, the relative use of NADH over NADPH are presented.

# Supplementary Material 8: Calculating the cytoplasmic acetoacetyl-CoA reductase and metabolites concentrations

Budde and co-workers measured the specific acetoacetyl-CoA reductase activity in different strains of *C. necator*, using AcAcCoA 32 µM and NADPH 100 µM ^16^. In the case of the *C. necator* strain Re2114 (Δ*phaB2* Δ*phaB3*), they observed an activity of 3.3 ± 0.4 U/mg. Considering that 68 % of the cell dry weight are proteins ^17^, 80 % of the cellular proteins are cytoplasmic ^18^ and a cytoplasmic volume of 1.9 mL_cytoplasm_/gCDW ^19^, it is possible to express the specific activity (*v*) in µmol^product^/L_cyt_/s. Moreover, considering *k_cat_* = 71 s^-1^, K_M_^AcAcCoA^ = K_i_^AcAcCoA^ = 2 µM ^20^; and K_M_^NADPH^ = 44 µM, K_iS_^AcAcCoA^ = 35 µM (this study); it is possible to calculate the cytoplasmic AAR^Cn1^ using the equation:

$$E=\frac{{v* K}_{M}^{AcAcCoA}*K_{M}^{NADPH}+K_{M}^{AcAcCoA}*NADPH +K_{M}^{NADPH}*AcAcCoA*\left( 1+\frac{AcAcCoA}{K_{iS}^{AcAcCoA}} \right)+AcAcCoA*NADPH}{k_{cat}^{NADPH}*NADPH*AcAcCoA}=134 {\mu mol}^{AARCn1}/L_{cytoplasm}$$

Cytoplasmic NAD(P)H concentration ranges were calculated as previously described ^21^. Briefly, it is possible to set and solve the following systems of algebraic equations:

For NAD(H):

NADH + NAD^+^ = 2650 µM ^22^ (I)

NADH/NAD^+^ = 0.03 or NADH/NAD^+^ = 0.71 ^23^ (II)

For NADP(H):

NADPH + NADP^+^ = 137 µM ^22^ (I)

NADPH/NADP^+^ = 0.32 ^24^ or NADPH/NADP^+^ = 60 ^22^ (II)

Cytoplasmic AcAcCoA concentrations were calculated such as the reaction catalyzed by β-ketothiolase be thermodynamically feasible. Considering R = 0.00831 kJ/K/mol, T = 310.15 K (37°C), Δ_r_G° = 25 kJ/mol - 1.7 kJ/mol (lower bound according to eQuilibrator ^25^), acetyl-CoA = 2 mM (upper bound according to Bennett and co-workers ^22^) and coenzyme A = 88 µM (lower bound according to Bennett and co-workers ^22^), it was possible to obtain the free Gibbs energy values of -4.36 kJ/mol, -2.57 kJ/mol and -1.52 kJ/mol for AcAcCoA concentrations of 1 µM, 2 µM and 3 µM.

For the sake of simplicity, the cytoplasmic concentration of 3-hydroxybutyryl-CoA (3HBCoA) was considered equivalent to the K_M_^3HBCoA^ = 15 µM (K_M_^(DL)-3HBCoA^ = 30 µM) observed for the NADPH-preferring acetoacetyl-CoA reductase studied by Haywood and co-workers ^11^. According to Bennett and co-workers, the K_M_ values of many metabolites reflect their cytoplasmic concentrations ^22^.

# Supplementary References

1 Bradford, M. M. A rapid and sensitive method for the quantitation of microgram quantities of protein utilizing the principle of protein-dye binding. *Analytical Biochemistry* **72**, 248-254, doi:10.1016/0003-2697(76)90527-3 (1976).

2 Wilkins, M. R. *et al.* Protein identification and analysis tools in the ExPASy server. *Methods Mol Biol* **112**, 531-552, doi:10.1385/1-59259-584-7:531 (1999).

3 Selwyn, M. J. A simple test for inactivation of an enzyme during assay. *Biochim Biophys Acta* **105**, 193-195 (1965).

4 Stern, J. R. Optical properties of aceto-acetyl-S-coenzyme A and its metal chelates. *J Biol Chem* **221**, 33-44 (1956).

5 Cornish-Bowden, A. *Fundamentals of Enzyme Kinetics*. 2nd edition edn, 344 (Portland Press, 1995).

6 Segel, I. H. *Enzyme kinetics: behavior and analysis of rapid equilibrium and steady state enzyme system*. (John Wiley & Sons,Incorporated, 1975).

7 Cornish-Bowden, A. The origins of enzyme kinetics. *FEBS Lett* **587**, 2725-2730, doi:10.1016/j.febslet.2013.06.009 (2013).

8 Ritchie, R. J. & Prvan, T. Current statistical methods for estimating the Km and Vmax of Michaelis-Menten kinetics. *Biochemical Education* **24**, 196-206, doi:<https://doi.org/10.1016/S0307-4412(96)00089-1> (1996).

9 Rohwer, J. M., Hanekom, A. J., Crous, C., Snoep, J. L. & Hofmeyr, J. H. Evaluation of a simplified generic bi-substrate rate equation for computational systems biology. *Syst Biol (Stevenage)* **153**, 338-341, doi:10.1049/ip-syb:20060026 (2006).

10 Schnell, S. & Mendoza, C. Closed Form Solution for Time-dependent Enzyme Kinetics. *Journal of Theoretical Biology* **187**, 207-212, doi:<https://doi.org/10.1006/jtbi.1997.0425> (1997).

11 Haywood, G. W., Anderson, A. J., Chu, L. & Dawes, E. A. The role of NADH- and NADPH-linked acetoacetyl-CoA reductases in the poly-3-hydroxybutyrate synthesizing organism Alcaligenes eutrophus FEMS Microbiology Letters Volume 52, Issue 3. *FEMS Microbiology Letters* **52**, 259-264 (1988). <<http://onlinelibrary.wiley.com/doi/10.1111/j.1574-6968.1988.tb02607.x/abstract>>.

12 Matsumoto, K. i. *et al.* Directed Evolution and Structural Analysis of NADPH-Dependent Acetoacetyl Coenzyme A (Acetoacetyl-CoA) Reductase from Ralstonia eutropha Reveals Two Mutations Responsible for Enhanced Kinetics. *Applied and environmental microbiology* **79**, 6134-6139, doi:10.1128/aem.01768-13 (2013).

13 Cavalieri, R. L. & Sable, H. Z. Pitfalls in the study of steady state kinetics of enzymes: spurious inhibition patterns due to stray light errors. *Anal Biochem* **59**, 122-128 (1974).

14 Ritchie, G. A., Senior, P. J. & Dawes, E. A. The purification and characterization of acetoacetyl-coenzyme A reductase from Azotobacter beijerinckii. *Biochem J* **121**, 309-316 (1971).

15 Kuzmic, P. Program DYNAFIT for the analysis of enzyme kinetic data: Application to HIV proteinase. *Analytical Biochemistry* **237**, 260-273, doi:10.1006/abio.1996.0238 (1996).

16 Budde, C. F., Mahan, A. E., Lu, J., Rha, C. & Sinskey, A. J. Roles of multiple acetoacetyl coenzyme A reductases in polyhydroxybutyrate biosynthesis in Ralstonia eutropha H16. *J Bacteriol* **192**, 5319-5328, doi:10.1128/jb.00207-10 (2010).

17 Taymaz-Nikerel, H., Borujeni, A. E., Verheijen, P. J., Heijnen, J. J. & van Gulik, W. M. Genome-derived minimal metabolic models for Escherichia coli MG1655 with estimated in vivo respiratory ATP stoichiometry. *Biotechnol Bioeng* **107**, 369-381, doi:10.1002/bit.22802 (2010).

18 Almen, M. S., Nordstrom, K. J., Fredriksson, R. & Schioth, H. B. Mapping the human membrane proteome: a majority of the human membrane proteins can be classified according to function and evolutionary origin. *BMC biology* **7**, 50, doi:10.1186/1741-7007-7-50 (2009).

19 Wang, L., Zhou, Y. J., Ji, D. & Zhao, Z. K. An accurate method for estimation of the intracellular aqueous volume of Escherichia coli cells. *Journal of Microbiological Methods* **93**, 73-76, doi:10.1016/j.mimet.2013.02.006 (2013).

20 Zhang, M., Kurita, S., Orita, I., Nakamura, S. & Fukui, T. Modification of acetoacetyl-CoA reduction step in Ralstonia eutropha for biosynthesis of poly(3-hydroxybutyrate-co-3-hydroxyhexanoate) from structurally unrelated compounds. *Microbial cell factories* **18**, 147, doi:10.1186/s12934-019-1197-7 (2019).

21 Olavarria, K. *et al.* An NADH preferring acetoacetyl-CoA reductase is engaged in poly-3-hydroxybutyrate accumulation in Escherichia coli. *J Biotechnol* **325**, 207-216, doi:10.1016/j.jbiotec.2020.10.022 (2021).

22 Bennett, B. D. *et al.* Absolute metabolite concentrations and implied enzyme active site occupancy in Escherichia coli. *Nat Chem Biol* **5**, 593-599, doi:10.1038/nchembio.186 (2009).

23 de Graef, M. R., Alexeeva, S., Snoep, J. L. & Teixeira de Mattos, M. J. The steady-state internal redox state (NADH/NAD) reflects the external redox state and is correlated with catabolic adaptation in Escherichia coli. *J Bacteriol* **181**, 2351-2357, doi:10.1128/JB.181.8.2351-2357.1999 (1999).

24 Chassagnole, C., Noisommit-Rizzi, N., Schmid, J. W., Mauch, K. & Reuss, M. Dynamic modeling of the central carbon metabolism of Escherichia coli. *Biotechnology and Bioengineering* **79**, 53-73, doi:10.1002/bit.10288 (2002).

25 Flamholz, A., Noor, E., Bar-Even, A. & Milo, R. eQuilibrator--the biochemical thermodynamics calculator. *Nucleic Acids Res* **40**, D770-775, doi:10.1093/nar/gkr874 (2012).

# Appendix 1: MATLAB code of the Simulation tool to evaluate accuracy of the expected kinetic parameters obtained from reaction progress curves analyses

clear

clc

disp('This script shows a comparison between the true kinetic parameters of a reversible BiBi reaction')

disp('and the kinetic parameter obtained, by a curve fitting procedure, under the assumption that such reaction is')

disp('an irreversible UniUni reaction (simple Michaelis-Menten)')

disp(' ')

disp('The reaction progress curves of reversible BiBi reactions are generated with the generalized BiBi equation proposed by Rohwer and co-workers in 2016')

disp('See the paper "Evaluation of a simplified generic bi-substrate rate equation for computational systems biology", by Rowher and co-workers ')

disp('DOI: 10.1049/ip-syb:20060026')

disp(' ')

disp('The user can define different time windows for the record of the reaction progress curves')

disp('In this example, we explored 10 time windows, from 3 to 30 minutes')

disp(' ')

disp('In this example, we explored 8 different combinations of enzyme concentration, KM of the substrate whose concentration is varied, and turnover constant (kcat_forward)')

disp('For each combination of enzyme concentration, KM and kcat_forward, the simulation tool will calculate the temporal changes in the concentrations of the substrates and products')

disp('The time vector and the product concentration vectors of each time window are the input for the estimation of KM and kcat, using the analytical solution of the Michaelis-Menten equation')

disp('See the paper: "Closed Form Solution for Time-dependent Enzyme Kinetics", Journal of Theoretical Biology. 187, 207-212.')

disp('https://doi.org/10.1006/jtbi.1997.0425')

%Defining the length of the experimental records (time windows, in minutes)

time_windows=3:3:30;

%Empty vectors to be filled with simulated values

kcat_values = zeros(length(time_windows),1);

KM_values = zeros(length(time_windows),1);

kcat_percents = zeros(length(time_windows),1);

KM_percents = zeros(length(time_windows),1);

fig = figure;

for z=1:8

% First, we need to "read" the kinetic parameters

[E,kcatf,KmA,KmB,KmP,KmQ,Keq]=BiBiparameters(z);

% Second, the script gets the initial concentrations of substrates and products

[concentrations_A,Bo]=InitialConcentrations;

for w=1:length(time_windows)

initial_time = 0; % initial time

final_time = time_windows(w)*60; % final time in seconds

integration_step = 1; % in seconds

time_vector = initial_time:integration_step:final_time;

initial_substrate_concentrations = [];

all_times = [];

all_P_in_t = [];

% In the following loop, the pseudoexperimental progress curves are generated

for i=1:length(concentrations_A)

[t,concentrations] = ode23tb(@(t,y) odeGenericBiBi(t,y,z),time_vector,[concentrations_A(i) Bo 0 0]);

initial_substrate_concentrations = [initial_substrate_concentrations repelem(concentrations_A(i),length(t))'];

all_times = [all_times t];

all_P_in_t = [all_P_in_t concentrations(:,3)];

end

% reading the time and product concentrations

data_adquisition_rate = 8; % time between the recorded experimental points, in seconds

experimental_substrate_concentrations = initial_substrate_concentrations(1:data_adquisition_rate:end,:);

experimental_times = all_times(1:data_adquisition_rate:end,:);

experimental_P_in_t = all_P_in_t(1:data_adquisition_rate:end,:);

[rows,columns]=size(experimental_P_in_t);

%Adding 5% Gaussian-distributed error to the pseudo-experimental curves

a = experimental_P_in_t;

sigmas = 0.05 * a;

randomNoise = randn(rows,columns) .* sigmas;

experimental_P_in_t = a + randomNoise;

% Setting the global fitting problem

p1 = repelem(E,rows*columns)'; % p1 is parameter 1, in this case enzyme concentration

p2 = experimental_substrate_concentrations(:); % p2 is parameter 2, in this case substrate concentration

x = experimental_times(:); % time values are the independent variable

y = experimental_P_in_t(:); % product concentration is the dependent variable

% Using the closed form solution Michaelis-Menten equation as the model to be fitted

f = fittype('(p2-KM*lambertw((p2/KM)*exp((-1*kcat*p1*x+p2)/(KM))))','coefficients',{'kcat','KM'},'problem',{'p1','p2'});

[A] = fit(x,y,f,'problem',{p1,p2},'StartPoint', [45,35]); % between square brackets, the initial guess for kcat and KM

coeffs = coeffvalues(A); kcat_best_fit=coeffs(1,1); KMA_best_fit=coeffs(1,2);

kcat_values(w)=kcat_best_fit;

KM_values(w)=KMA_best_fit;

kcat_percents(w) = (kcat_best_fit/kcatf)*100;

KM_percents(w) = (KMA_best_fit/KmA)*100;

end

subplot(4,2,z)

scatter (time_windows,KM_percents,'r','filled');

x1 = time_windows;

y1 = KM_percents;

x2 = time_windows;

y2 = kcat_percents;

ax1 = gca;

set(ax1,'XColor','k','YColor','r');

ax2 = axes('Position',get(ax1,'Position'),...

'XAxisLocation','bottom',...

'YAxisLocation','right',...

'Color','none',...

'XColor','none','YColor','b');

hold on

scatter (time_windows,kcat_percents,'b','filled');

txt1 = strcat('K_M=',num2str(KmA),'\muM ','{\it k_c_a_t}=',num2str(kcatf),'s^-^1','E=',num2str(E),'\muM');

title(txt1);

ax = gca;

ax.TitleFontSizeMultiplier = 0.8;

set(gcf,'color','w');

hold on;

han=axes(fig,'visible','off');

ax2 = axes('Position',get(han,'Position'),...

'XAxisLocation','bottom',...

'YAxisLocation','right',...

'Color','none',...

'XColor','none','YColor','k','visible','off');

han.XLabel.Visible='on';

han.YLabel.Visible='on';

ax2.XLabel.Visible='on';

ax2.YLabel.Visible='on';

ylabel(han,'deviation from "true" {\it k_{cat}} (%)','color','r');

xlabel(han,'recording time (min)');

ylabel(ax2,'deviation from "true" K_M (%)','color','b');

end

function dy = odeGenericBiBi(~,y,z)

[E,kcatf,KmA,KmB,KmP,KmQ,Keq]=BiBiparameters(z);

Vmax = E * kcatf;

dy = zeros(4,1);

dy(1)=-1*((1-((y(4)*y(3))/(Keq*y(1)*y(2))))*((Vmax*y(1)*y(2))/(KmA*KmB)))/((1+y(1)/KmA+y(3)/KmP)*(1+y(2)/KmB+y(4)/KmQ));

dy(2)=-1*((1-((y(4)*y(3))/(Keq*y(1)*y(2))))*((Vmax*y(1)*y(2))/(KmA*KmB)))/((1+y(1)/KmA+y(3)/KmP)*(1+y(2)/KmB+y(4)/KmQ));

dy(3)= ((1-((y(4)*y(3))/(Keq*y(1)*y(2))))*((Vmax*y(1)*y(2))/(KmA*KmB)))/((1+y(1)/KmA+y(3)/KmP)*(1+y(2)/KmB+y(4)/KmQ));

dy(4)= ((1-((y(4)*y(3))/(Keq*y(1)*y(2))))*((Vmax*y(1)*y(2))/(KmA*KmB)))/((1+y(1)/KmA+y(3)/KmP)*(1+y(2)/KmB+y(4)/KmQ));

end

function [E,kcatf,KmA,KmB,KmP,KmQ,Keq]=BiBiparameters(z)

Evalues=[0.001 0.001 0.001 0.001 0.004 0.004 0.004 0.004];

KmAvalues=[30 30 3000 3000 30 30 3000 3000];

kcatfvalues=[5 50 5 50 5 50 5 50];

E = Evalues(z);

Keq = 92;

kcatf = kcatfvalues(z);

KmA = KmAvalues(z);

KmB = 20;

KmP = 10*KmA;

KmQ = 300;

end

function [concentrations_A,Bo]=InitialConcentrations

concentrations_A = [10 20 25 30 40 50 70 80 100 120 140 170 200 230 260 290 320 360 400 440 480 520 560 600];

Bo = 2000;

end

# Appendix 2: MATLAB code to calculate the flux capacity and cofactor preference of AAR^Cn1^ and Chimera 5

clc

clear

% Thermodynamic and cellular parameters

R = (8.31446261815324/1000); TCelsius = 37; T = 273.15 + TCelsius;

dGst_thiolase = 25-1.7; dGst_AAR = -14.3-7.1;

Keq_thiolase = exp(-1*dGst_thiolase/R/T); Keq_AAR = exp(-1*dGst_AAR/R/T);

cyt_volume = 526; fraction_cyt_prots = 0.8; fraction_prots_BM = 0.68;

conv_factor1 = 3600/1000/cyt_volume;

AcCoA = 2000; CoA = 88; HBCoA = 15;

% AcAcCoA concentrations enabling a thermodynamically feasible beta-ketothiolase-catalyzed reaction

AcAcCoA = [1 2 3];

cofactor_points = 50; %if a more detailed description is wanted, it is possible to add more cofactor points

% Data from Budde et al (2010): doi:10.1128/JB.00207-10

AcAcCoA_assay = 32; NADPH_assay = 100; %substrate concentrations in microM

spAARCn = 3.3; % specific AAR activity in U/mg

%Kinetic parameters of AARCn1

AARCnkcatNADPH = 71;

AARCnKMNADPH = 44;

AARCnKMAcAcCoANADPH = 2;

AARCnKiAcAcCoANADPH = AARCnKMAcAcCoANADPH;

AARCnKiSAcAcCoANADPH = 35;

AARCnkcatNADH = AARCnkcatNADPH*0.2; %According to Haywood et al.(1988) https://www.sciencedirect.com/science/article/pii/0378109788903722

AARCnKMNADH = 819;

AARCnKMAcAcCoANADH = AARCnKMAcAcCoANADPH;

AARCnKiAcAcCoANADH = AARCnKMAcAcCoANADH;

AARCnKiSAcAcCoANADH = Inf; %No substrate inhibition so far described in this case

%Calculating enzyme concentration using kinetic parameters and data from

%measured specific activity in cell-free extract

E = (fraction_prots_BM*fraction_cyt_prots*cyt_volume*1000/60)*(spAARCn*AARCnKiAcAcCoANADPH*AARCnKMNADPH+AARCnKMAcAcCoANADPH*NADPH_assay+AARCnKMNADPH*AcAcCoA_assay*(1+AcAcCoA_assay/AARCnKiSAcAcCoANADPH)+AcAcCoA_assay*NADPH_assay)/(AARCnkcatNADPH*AcAcCoA_assay*NADPH_assay);

%Kinetic parameters of Chimera 5

AARCh5kcatNADPH = 76;

AARCh5KMNADPH = 2787;

AARCh5KMAcAcCoANADPH = 48;

AARCh5KiAcAcCoANADPH = AARCh5KMAcAcCoANADPH;

AARCh5KiSAcAcCoANADPH = 324;

AARCh5kcatNADH = 147;

AARCh5KMNADH = 43;

AARCh5KMAcAcCoANADH = 50;

AARCh5KiAcAcCoANADH = AARCh5KMAcAcCoANADH;

AARCh5KiSAcAcCoANADH = AARCh5KMAcAcCoANADH;

%Calculating cofactor concentration ranges

moiety_size_NAD = 2650; %Bennett BD, Kimball EH, Gao M, Osterhout R, Van Dien SJ, Rabinowitz JD. Absolute metabolite concentrations and implied enzyme active site occupancy in Escherichia coli. Nat Chem Biol. 2009;5(8):593-599. doi:10.1038/nchembio.186

NADH_over_NAD_ratio_oxidized = 0.03; %de Graef, M. R., Alexeeva, S., Snoep, J. L., Teixeira de Mattos, M. J., The steady-state internal redox state (NADH/NAD) reflects the external redox state and is correlated with catabolic adaptation in Escherichia coli. J Bacteriol 1999, 181, 2351-2357.

NADH_over_NAD_ratio_reduced = 0.71; %de Graef, M. R., Alexeeva, S., Snoep, J. L., Teixeira de Mattos, M. J., The steady-state internal redox state (NADH/NAD) reflects the external redox state and is correlated with catabolic adaptation in Escherichia coli. J Bacteriol 1999, 181, 2351-2357.

moiety_size_NADP = 137; %Bennett BD, Kimball EH, Gao M, Osterhout R, Van Dien SJ, Rabinowitz JD. Absolute metabolite concentrations and implied enzyme active site occupancy in Escherichia coli. Nat Chem Biol. 2009;5(8):593-599. doi:10.1038/nchembio.186

NADPH_over_NADP_ratio_oxidized = 0.32; %Chassagnole C, Noisommit-Rizzi N, Schmid JW, Mauch K, Reuss M. Dynamic modeling of the central carbon metabolism of Escherichia coli. Biotechnol Bioeng. 2002;79(1):53-73. doi:10.1002/bit.10288

NADPH_over_NADP_ratio_reduced = 60; %Bennett BD, Kimball EH, Gao M, Osterhout R, Van Dien SJ, Rabinowitz JD. Absolute metabolite concentrations and implied enzyme active site occupancy in Escherichia coli. Nat Chem Biol. 2009;5(8):593-599. doi:10.1038/nchembio.186

%%%%%%%%%%%%%%%

syms NAD_oxidized NADH_oxidized

[NAD_oxidized,NADH_oxidized] = solve(NAD_oxidized + NADH_oxidized==moiety_size_NAD , NADH_oxidized/NAD_oxidized==NADH_over_NAD_ratio_oxidized);

syms NAD_reduced NADH_reduced

[NAD_reduced,NADH_reduced] = solve(NAD_reduced + NADH_reduced==moiety_size_NAD , NADH_reduced/NAD_reduced==NADH_over_NAD_ratio_reduced);

%%%%%%%%%%%%%%%

syms NADP_oxidized NADPH_oxidized

[NADP_oxidized,NADPH_oxidized] = solve(NADP_oxidized + NADPH_oxidized==moiety_size_NADP , NADPH_oxidized/NADP_oxidized==NADPH_over_NADP_ratio_oxidized);

syms NADP_reduced NADPH_reduced

[NADP_reduced,NADPH_reduced] = solve(NADP_reduced + NADPH_reduced==moiety_size_NADP , NADPH_reduced/NADP_reduced==NADPH_over_NADP_ratio_reduced);

%%%%%%%%%%%%%%%

NAD = flip(double(linspace(NAD_reduced,NAD_oxidized,cofactor_points))');

NADH = flip(double(linspace(NADH_reduced,NADH_oxidized,cofactor_points))');

NADP = flip(double(linspace(NADP_reduced,NADP_oxidized,cofactor_points))');

NADPH = flip(double(linspace(NADPH_reduced,NADPH_oxidized,cofactor_points))');

for i=1:length(NADH)

ratioNADH(i) = NADH(i)/NAD(i);

ratioNADPH(i) = NADPH(i)/NADP(i);

end

ratioNADH = ratioNADH';

ratioNADPH = ratioNADPH';

dGr_thiolase = zeros(length(AcAcCoA),1);

dGr_AAR_NAD = zeros(cofactor_points,length(AcAcCoA));

dGr_AAR_NADP = zeros(cofactor_points,length(AcAcCoA));

Marker_Counter = 1;

Markers = {'+k','ok','*k','xk','vk','dk'};

mks = 10; %marker size

fts = 10; %font size

for i=1:length(AcAcCoA)

dGr_thiolase(i) = dGst_thiolase + R*T*log((AcAcCoA(i)*CoA)/(AcCoA^2));

for m=1:cofactor_points

dGr_AAR_NAD(m,i) = dGst_AAR + R*T*log((NAD(m)*HBCoA)/(NADH(m)*AcAcCoA(i)));

dGr_AAR_NADP(m,i) = dGst_AAR + R*T*log((NADP(m)*HBCoA)/(NADPH(m)*AcAcCoA(i)));

vAARCnNADPH_f(m,i) = conv_factor1*(E*AARCnkcatNADPH*AcAcCoA(i)*NADPH(m))/(AARCnKiAcAcCoANADPH*AARCnKMNADPH+AARCnKMAcAcCoANADPH*NADPH(m)+AARCnKMNADPH*AcAcCoA(i)*(1+AcAcCoA(i)/AARCnKiSAcAcCoANADPH)+AcAcCoA(i)*NADPH(m));

vAARCnNADPH_r(m,i) = vAARCnNADPH_f(m,i)*exp(dGr_AAR_NADP(m,i)/(R*T));

vAARCnNADPH_net(m,i) = vAARCnNADPH_f(m,i)-vAARCnNADPH_r(m,i);

vAARCnNADH_f(m,i) = conv_factor1*(E*AARCnkcatNADH*AcAcCoA(i)*NADH(m))/(AARCnKiAcAcCoANADH*AARCnKMNADH+AARCnKMAcAcCoANADH*NADH(m)+AARCnKMNADH*AcAcCoA(i)*(1+AcAcCoA(i)/AARCnKiSAcAcCoANADH)+AcAcCoA(i)*NADH(m));

vAARCnNADH_r(m,i) = vAARCnNADH_f(m,i)*exp(dGr_AAR_NAD(m,i)/(R*T));

vAARCnNADH_net(m,i) = vAARCnNADH_f(m,i)-vAARCnNADH_r(m,i);

vAARCh5NADPH_f(m,i) = conv_factor1*(E*AARCh5kcatNADPH*AcAcCoA(i)*NADPH(m))/(AARCh5KiAcAcCoANADPH*AARCh5KMNADPH+AARCh5KMAcAcCoANADPH*NADPH(m)+AARCh5KMNADPH*AcAcCoA(i)*(1+AcAcCoA(i)/AARCh5KiSAcAcCoANADPH)+AcAcCoA(i)*NADPH(m));

vAARCh5NADPH_r(m,i) = vAARCh5NADPH_f(m,i)*exp(dGr_AAR_NADP(m,i)/(R*T));

vAARCh5NADPH_net(m,i) = vAARCh5NADPH_f(m,i)-vAARCh5NADPH_r(m,i);

vAARCh5NADH_f(m,i) = conv_factor1*(E*AARCh5kcatNADH*AcAcCoA(i)*NADH(m))/(AARCh5KiAcAcCoANADH*AARCh5KMNADH+AARCh5KMAcAcCoANADH*NADH(m)+AARCh5KMNADH*AcAcCoA(i)*(1+AcAcCoA(i)/AARCh5KiSAcAcCoANADH)+AcAcCoA(i)*NADH(m));

vAARCh5NADH_r(m,i) = vAARCh5NADH_f(m,i)*exp(dGr_AAR_NAD(m,i)/(R*T));

vAARCh5NADH_net(m,i) = vAARCh5NADH_f(m,i)-vAARCh5NADH_r(m,i);

end

%%%%%%%%%%%%%%%%%%%%%%%%%%%%%%%%%%%%%%%%%%%%%%%%%%%%%%

figure(1)

txt = ['AcAcCoA=',num2str(AcAcCoA(i)),'\muM',' \Delta_rG^{thiolase}=',num2str(round((dGr_thiolase(i))*100)/100),' kJ/mol'];

subplot(2,2,1)

scatter(NADH,vAARCnNADH_net(:,i),mks,Markers{Marker_Counter},'DisplayName',txt);

title('AAR^{Cn1} using NADH')

xlabel('NADH (\muM)')

ylabel('J^{net} (mmol/g_{CDW}/h^{-1})')

set(gca,'FontSize',fts)

hold on

subplot(2,2,2)

scatter(NADPH,vAARCnNADPH_net(:,i),mks,Markers{Marker_Counter},'DisplayName',txt);

title('AAR^{Cn1} using NADPH')

xlabel('NADPH (\muM)')

ylabel('J^{net} (mmol/g_{CDW}/h^{-1})')

set(gca,'FontSize',fts)

hold on

subplot(2,2,3)

scatter(NADH,vAARCh5NADH_net(:,i),mks,Markers{Marker_Counter},'DisplayName',txt);

title('Chimera 5 using NADH')

xlabel('NADH (\muM)')

ylabel('J^{net} (mmol/g_{CDW}/h^{-1})')

set(gca,'FontSize',fts)

hold on

subplot(2,2,4)

scatter(NADPH,vAARCh5NADPH_net(:,i),mks,Markers{Marker_Counter},'DisplayName',txt);

title('Chimera 5 using NADPH')

xlabel('NADPH (\muM)')

ylabel('J^{net} (mmol/g_{CDW}/h^{-1})')

set(gca,'FontSize',fts)

hold on

set(gcf,'color','w');

Marker_Counter = Marker_Counter+1;

end

legend ('Interpreter','tex')

legend boxoff

for j=1:cofactor_points

for n=1:cofactor_points

NADHmatrix(j,n) = NADH(n);

NADPHmatrix(j,n) = NADPH(j);

AARCnpreferences(j,n) = vAARCnNADPH_net(j,i)/vAARCnNADH_net(n,i);

AARCh5preferences(j,n) = vAARCh5NADH_net(n,i)/vAARCh5NADPH_net(j,i);

end

end

figure(2)

subplot(1,2,1)

surf(NADHmatrix,NADPHmatrix,AARCnpreferences);

title('AAR^{Cn1}');

xlabel('NADH (\muM)');

ylabel('NADPH (\muM)');

zlabel('J^{net,NADPH}/J^{net,NADH}');

set(gca,'FontSize',12);

set(gcf,'color','w');

colorbar('northoutside');

subplot(1,2,2)

surf(NADHmatrix,NADPHmatrix,AARCh5preferences);

title('Chimera 5');

xlabel('NADH (\muM)');

ylabel('NADPH (\muM)');

zlabel('J^{net,NADH}/J^{net,NADPH}');

set(gca,'FontSize',12);

set(gcf,'color','w');

colorbar('northoutside');
